# FUS Interacts with HSP60 to Promote Mitochondrial Damage

**DOI:** 10.1371/journal.pgen.1005357

**Published:** 2015-09-03

**Authors:** Jianwen Deng, Mengxue Yang, Yanbo Chen, Xiaoping Chen, Jianghong Liu, Shufeng Sun, Haipeng Cheng, Yang Li, Eileen H. Bigio, Marsel Mesulam, Qi Xu, Sidan Du, Kazuo Fushimi, Li Zhu, Jane Y. Wu

**Affiliations:** 1 State Key Laboratory of Brain and Cognitive Science, Institute of Biophysics, Chinese Academy of Sciences, Beijing, China; 2 University of Chinese Academy of Sciences, Beijing, China; 3 Department of Neurology, Center for Genetic Medicine, Lurie Cancer Center, Northwestern University Feinberg School of Medicine, Chicago, Illinois, United States of America; 4 National Laboratory of Medical Molecular Biology, Institute of Basic Medical Sciences, Chinese Academy of Medical Science and Peking Union Medical College, Tsinghua University, Beijing, China; 5 School of Electronic Science and Engineering, Nanjing University, Nanjing, China; 6 Department of Pathology & Neurology, The Cognitive Neurology& Alzheimer's Disease Center, Northwestern University Feinberg School of Medicine, Chicago, Illinois, United States of America; Stanford University School of Medicine, UNITED STATES

## Abstract

FUS-proteinopathies, a group of heterogeneous disorders including ALS-FUS and FTLD-FUS, are characterized by the formation of inclusion bodies containing the nuclear protein FUS in the affected patients. However, the underlying molecular and cellular defects remain unclear. Here we provide evidence for mitochondrial localization of FUS and its induction of mitochondrial damage. Remarkably, FTLD-FUS brain samples show increased FUS expression and mitochondrial defects. Biochemical and genetic data demonstrate that FUS interacts with a mitochondrial chaperonin, HSP60, and that FUS translocation to mitochondria is, at least in part, mediated by HSP60. Down-regulating HSP60 reduces mitochondrially localized FUS and partially rescues mitochondrial defects and neurodegenerative phenotypes caused by FUS expression in transgenic flies. This is the first report of direct mitochondrial targeting by a nuclear protein associated with neurodegeneration, suggesting that mitochondrial impairment may represent a critical event in different forms of FUS-proteinopathies and a common pathological feature for both ALS-FUS and FTLD-FUS. Our study offers a potential explanation for the highly heterogeneous nature and complex genetic presentation of different forms of FUS-proteinopathies. Our data also suggest that mitochondrial damage may be a target in future development of diagnostic and therapeutic tools for FUS-proteinopathies, a group of devastating neurodegenerative diseases.

## Author Summary

Amyotrophic lateral sclerosis (ALS) and frontotemporal lobar degeneration (FTLD) are two groups of common and devastating neurodegenerative diseases, characterized by losses of selected groups of neurons. Mutations in the FUS gene have been associated with ALS, whereas inclusion bodies containing the FUS protein have been discovered in both ALS and FTLD patients. However, the underlying pathogenic mechanisms of FUS in these diseases remain unclear. Here, we demonstrate that wild-type or ALS-associated mutant FUS can interact with mitochondrial chaperonin HSP60 and that HSP60 mediates FUS localization to mitochondria, leading to mitochondrial damage. Mitochondrial impairment may be an early event in FUS proteinopathies and represent a potential therapeutic target for treating these fatal diseases.

## Introduction

Amyotrophic lateral sclerosis (ALS) is a fatal neurodegenerative disease primarily affecting motor neurons. The Cu/Zn superoxide dismutase 1 (SOD1) gene was the first ALS-associated gene whose mutations were identified in familial ALS (fALS) patients [1,2]. Subsequently, genetic studies have uncovered more than ten ALS-associated genes [3,4,5]. Among these are genes encoding RNA/DNA binding proteins, including TAR-DNA binding protein of 43 kDa (TDP-43) and fused in sarcoma/translocated in liposarcoma (FUS/TLS or FUS) [6,7,8,9]. Pathologically, FUS immunoreactive inclusion bodies are detected in a range of neurological diseases classified as FUS-proteinopathies. These disorders are genetically and clinically heterogeneous. Depending on the regions affected, FUS-proteinopathies can manifest as motor neuron disease such as ALS-FUS, or as various forms of dementia including frontotemporal lobar degeneration with FUS pathology (FTLD-FUS),atypical FTLD with ubiquitin pathology (aFTLD-U), neuronal intermediate filament inclusion disease (NIFID), and Basophilic Inclusion Body Disease (BIBD) [4,10,11,12,13,14]. Interestingly, although >30 mutations in the FUS gene have been found in patients with ALS, no FUS mutations have been detected in the vast majority of sporadic or familial, pathologically proven cases of FTLD-FUS [15]. A recent study has identified several mutations in the 3’ untranslated region of the FUS gene that are associated with increased FUS expression among ALS patients [16], suggesting that increased FUS expression could be a mechanism contributing to the pathogenesis of ALS.

Several systems have been used to model FUS-proteinopathies, ranging from yeast to vertebrate animals [17,18,19,20,21,22,23,24,25]. A few groups, including Lanson and colleagues as well as our team, have established transgenic flies expressing wild-type (Wt) or ALS-mutant forms of human FUS protein [17,22]. In our transgenic fly model, targeted expression of either Wt- or ALS-mutant FUS protein in specific neuronal subpopulations leads to age-dependent neurodegeneration with functional deficits, recapitulating the critical features of FUS proteinopathies [17]. In transgenic mice, simply overexpressing the wild-type FUS led to progressive neurodegeneration [26]. Because no FUS mutation has been detected in most FTLD-FUS patients and because the patient samples that we examined showed elevated FUS protein levels (see below), the work in this study examining the effects of increased Wt-FUS expression is pertinent to understanding FTLD-FUS, whereas the data with ALS-mutant FUS, such as P525L, is relevant to ALS-FUS.

To understand the biological function of mammalian FUS in the nervous system, we searched for interaction partners of FUS. Using a FUS-specific monoclonal antibody (S1 Fig; also see [27]),we developed an immunopurification-coupled mass spectrometry approach to identify proteins that interact with FUS in the bovine brain tissue. Among candidate FUS interaction partners, several mitochondrial proteins were identified, including HSP60 (see below; detailed data to be reported in a separate study). Consistent with this finding, the endogenous FUS protein was detected by mass-spectrometry in biochemically-purified mitochondria (without using the FUS antibody or overexpressing FUS), supporting the idea that FUS interacts with mitochondria. These observations prompted us to carefully examine mitochondria in our models for FUS proteinopathies.

HSP60 proteins are a family of evolutionarily conserved ATP-dependent chaperones that play important roles in stress response, protein folding and cell signaling [28,29,30]. They are expressed constitutively as well as in response to stress signals [31,32]. HSP60 proteins are detected in the cytosol and inside the mitochondrial matrix. It has been reported that HSP60, together with other heat shock proteins such as HSP10 and HSP70, facilitates proper protein folding and assembly of protein complexes imported into mitochondria [30,32,33,34,35,36]. Mutations in the HSPD1 gene (encoding the human HSP60 protein) have been found in patients with spastic paraplegia type 13 (SPG13), a late-onset autosomal-dominant neurodegenerative disease characterized by progressive weakness and spasticity of lower limbs [37,38].

Mitochondrial impairment has been extensively investigated in ALS, in particular, in SOD1 animal models [39,40,41,42,43]. Aggregated mitochondria have been reported in transgenic mice overexpressing TDP-43 [44,45]. An EM study of spinal cord samples of two cases of ALS-FUS, including one containing P525L mutation, revealed disorganized mitochondria and endoplasmic reticulum [46]. Cytoplasmic expression of two other ALS-associated FUS mutants, R521G or R521H, was associated with shortened mitochondria in cultured motor neurons [47]. These studies suggest mitochondrial damage may be a common feature in ALS-FUS. However, no evidence has been reported for the mitochondrial localization of FUS or for mitochondrial damage in FTLD-FUS patients. Although it remains to be determined if mitochondrial impairment is a direct consequence of FUS expression, our data presented here show that mitochondrial fragmentation was detected not only *in vitro* in cultured neurons but also *in vivo* in motor neurons in the FUS-transgenic flies. Both Wt- and ALS-mutant P525L FUS interacted with HSP60.Furthermore, mitochondrial damage was detected in brain samples of FTLD-FUS patients, with FUS levels increased in all 3 FTLD-FUS patient brain samples examined. Remarkably, elevated HSP60 expression was detected in two of these 3 cases of FTLD-FUS patient brain samples. Knocking-down HSP60 led to reduced level of mitochondrial FUS in cultured cells. Interestingly, RNAi-mediated down-regulation of the HSP60 homolog partially rescued the neurodegenerative phenotypes in FUS transgenic flies. Thus, increased FUS expression and mitochondrial impairment appear as a prominent pathological feature in FTLD-FUS. Our data also uncover the previously unknown regulation of FUS subcellular distribution by chaperon proteins such as HSP60, suggesting a new direction for treating FUS proteinopathies by modulating mitochondrial localization of FUS and protecting against FUS-induced mitochondrial damage.

## Results

### Expression of Wt- or ALS-associated mutant FUS led to mitochondrial fragmentation in mammalian neuron-like cells, cultured neurons and FUS transgenic flies

**Fig 1:**
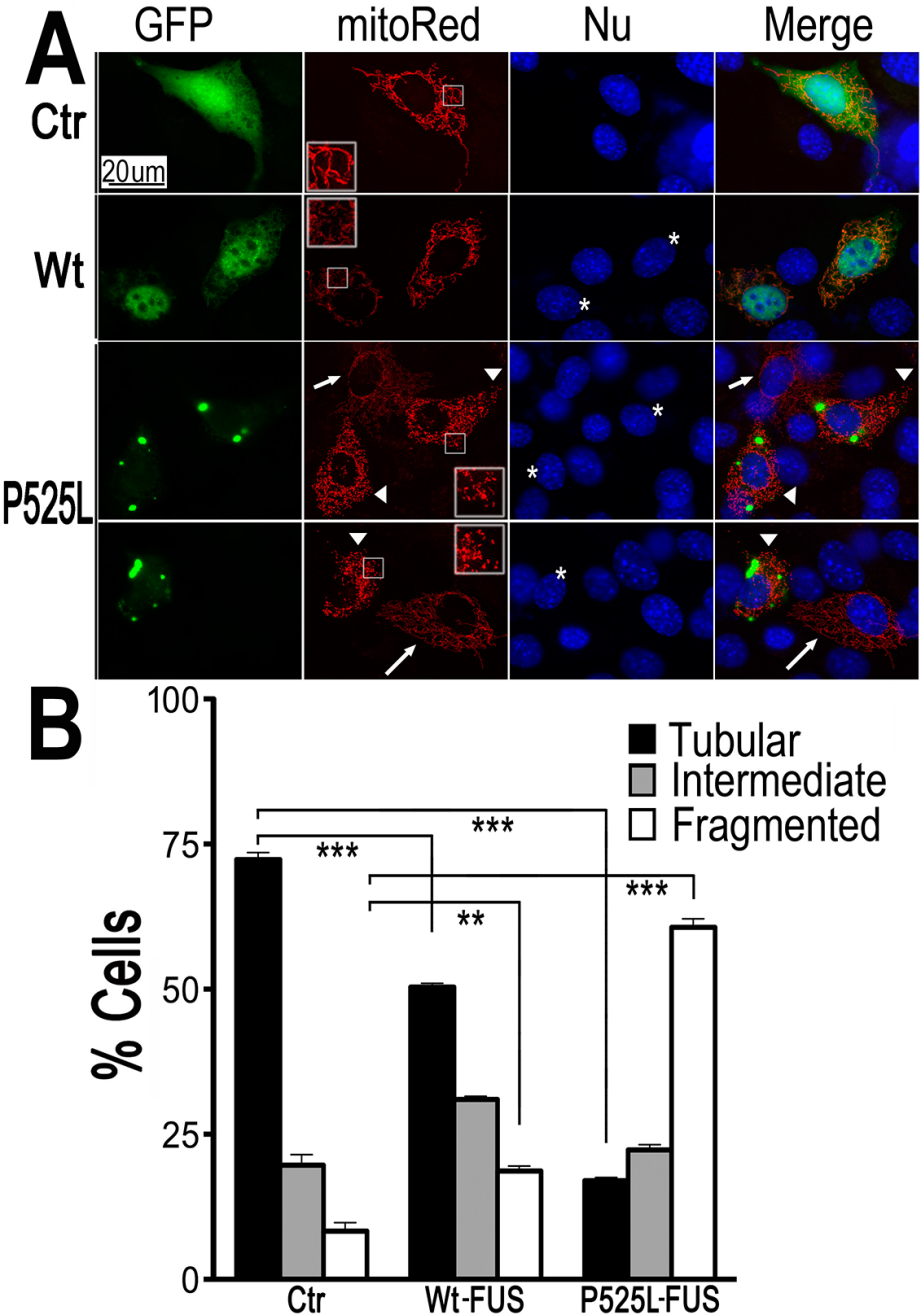
Expression of Wt- or ALS-mutant FUS in HT22 neuron-like cells led to mitochondrial fragmentation. **(A)**The mitoRed plasmid was co-transfected together with plasmids expressing either the control GFP vector (Ctr), or Wt- or P525L-mutant FUS into HT22 cells. Cells with tubular, intermediate or fragmented mitochondrial patterns were imaged 72 hrs post-transfection and quantified. Arrowheads mark the cells that expressed exogenous FUS and showed fragmented mitochondria (with their nuclei marked by “*”); whereas the arrows mark adjacent non-transfected cells showing tubular mitochondria. Insets show the boxed areas at a higher magnification.(B)Quantification of mitochondrial fragmentation from experiments shown in panel A. The data were analyzed using one-way ANOVA with Bonferroni post-test (**: p<0.001; ***: p<0.0001).

To examine if FUS expression affects mitochondria in mammalian cells, we expressed FUS in neuron-like cells, HT-22, an immortalized mouse cell line with neuronal features [48]. To monitor changes in the mitochondrial morphology, a plasmid expressing mitochondrion-targeted red fluorescent protein (RFP) (mito-Red) was co-transfected with either a GFP vector control or a plasmid expressing GFP-tagged FUS protein into HT-22 cells (Fig 1A). The majority of cells expressing GFP vector control showed mitochondria with tubular morphology (Fig 1A). However, the percentage of the cells with fragmented mitochondria was significantly increased when either Wt-FUS or the ALS-mutant P525L was expressed. This mitochondrial change was particularly pronounced in cells expressing the P525L-mutant FUS (Fig 1B).

**Fig 2:**
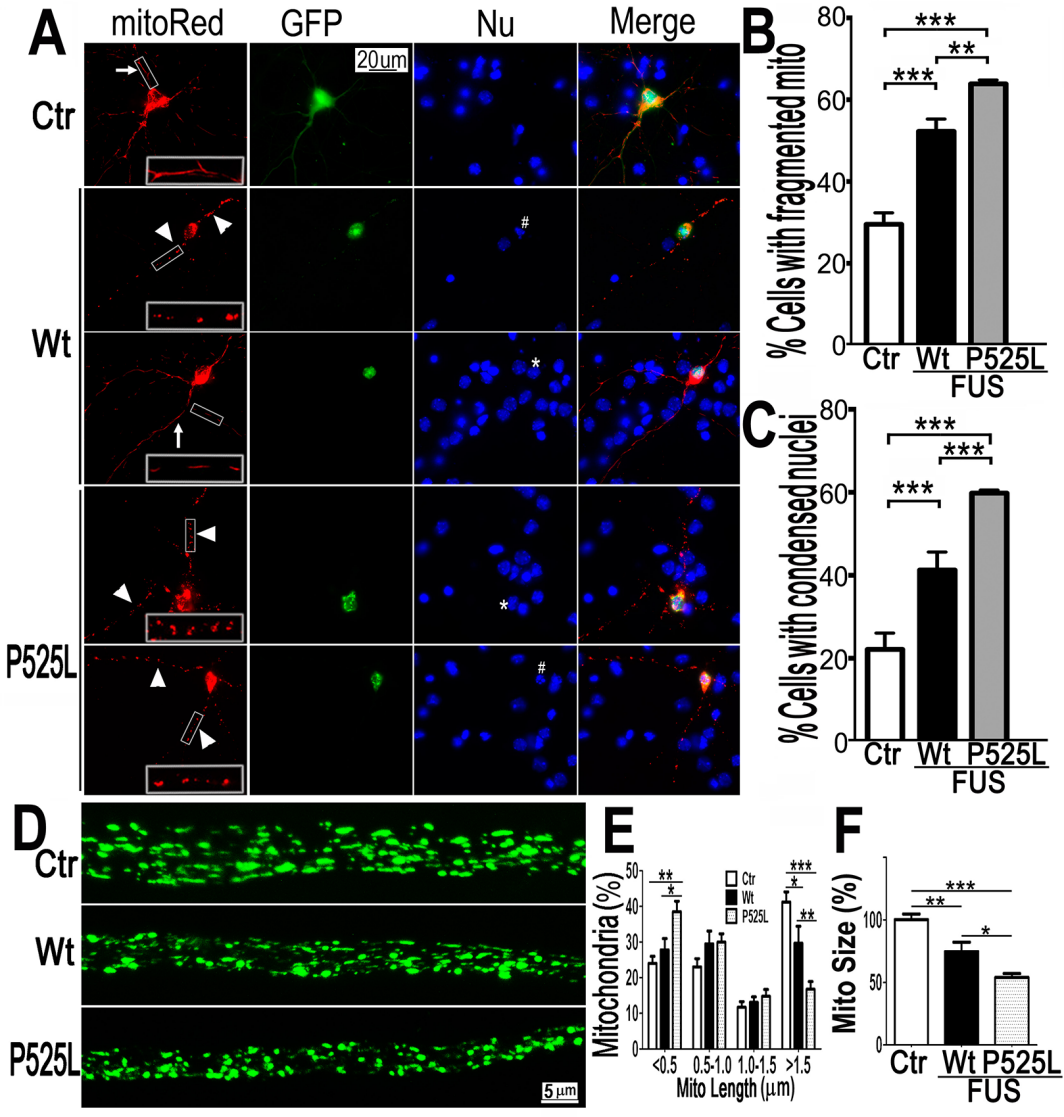
Expression of Wt- or P525L- mutant FUS in primary cortical neurons (A-C) or fly motor neurons (D-F)led to mitochondrial fragmentation. The mitoRed plasmid was co-transfected together with plasmids expressing either the control GFP vector (Ctr), or Wt- or P525L-mutant FUS into E18 murine cortical neurons(A-C)with quantification of apoptotic neurons containing condensed nuclei as previously published [94], shown in panel B and C respectively. In panel A, arrows label neurons showing normal mitochondria, whereas the arrowheads mark neurons showing fragmented mitochondria. The “*” labels nuclei with normal morphology, whereas “#” mark the condensed or fragmented nuclei (signs of apoptosis). The higher magnification images of the boxed areas are shown in insets at the bottom of the first set of images in panel A. (D-F). FUS expression in fly motor neurons (MNs) led to mitochondrial fragmentation. In the axonal bundles of MNs, mitochondria were visualized by mitoGFP expression in the 3rd instar larvae. (D) Confocal microscopic images of motor neuron axons in the A3 abdominal segment were obtained in Z-stacks and projected into single images, as described previously [90]. Fly genotypes, Ctr: D42-Gal4/UAS-mitoGFP/UAS-RFP; Wt: D42-Gal4/UAS-mitoGFP/UAS-Wt-FUS-RFP; P525L: D42-Gal4/UAS-mitoGFP/UAS-P525L-FUS-RFP. (E) Quantification of mitochondrial size distribution using Image J. The Wt- or P525L-mutant FUS expressing flies showed significantly increased numbers of smaller mitochondria, but fewer mitochondria with larger sizes as compared with the control group. Ten larvae were quantified in each group. (F) Quantification of mitochondrial lengths in MNs axons. The 3rd instar larvae expressing Wt- or P525L-mutant FUS showed significantly smaller mitochondria as compared with the control flies. Ten larvae were measured in each group. All data were analyzed using one-way ANOVA with Bonferronipost-test (*: p<0.05; **: p<0.01; ***: p<0.0001).

Next, mito-Red and FUS-GFP were co-expressed in cultured mouse cortical neurons. Fluorescent confocal microscopy revealed a significant increase in the percentage of neurons with fragmented mitochondria when expressing either Wt- or P525L-mutant FUS. The percentage of neurons expressing the P525L-mutant FUS showed fragmented mitochondria is higher than those expressing Wt-FUS (Fig 2A–2C). Some of these neurons expressing FUS also showed condensed or fragmented nuclei, a sign of cell death. It is important to note that in a significant fraction of FUS-expressing HT22 cells or cortical neurons that showed mitochondrial fragmentation, there was no detectable sign of nuclear morphological changes characteristic of cell death, suggesting that mitochondrial changes may be an early event, preceding cell death. These data indicate that increased expression of FUS, especially the P525L-mutant, promotes mitochondrial fragmentation and cell death in mammalian neurons.

To test *in vivo* effects of FUS expression, we examined motor neurons (MNs) expressing human FUS in transgenic flies [17]. The D42-Gal4 driver [49,50] was used to express either Wt- or P525L-mutant FUS specifically in *Drosophila* MNs. Mitochondria in MN axons were examined with confocal microscopy by the expression of mito-GFP in wandering 3rd instar larvae (Fig 2D). It should be noted that the flies expressing the P525L-mutant FUS used in this study were from a line with a less severe phenotype [17], because other P525L-mutant FUS lines exhibiting more severe phenotypes did not survive to the late larval stage. As compared with the control flies, axonal mitochondria were significantly smaller in MNs expressing the Wt- or P525L-mutant FUS protein. MNs expressing the human FUS protein showed an increase in the percentage of smaller mitochondria (length<0.5μm) and a decrease in the percentage of larger mitochondria (length>1.5μm, p<0.05), with P525L-mutant exhibiting more severe defects than the Wt-FUS (p<0.01; Fig 2E). Consistently, the average mitochondrial size in the FUS-expressing fly MNs were significantly smaller than that in the control group (p<0.01), with the P525L-mutant expressing flies showing further shortened average mitochondrial length than the Wt- group (p<0.05; Fig 2F). These data indicate that the expression of either Wt- or P525L-mutant FUS induces mitochondrial defects *in vivo*, with the ALS-mutant eliciting a more severe phenotype. This is consistent with our previous observation that neurodegeneration phenotypes in flies expressing the P525L-mutant were more severe than those expressing Wt-FUS [17].

Interestingly, cells transfected with either the Wt- or P525L-mutant FUS consistently showed a moderate increase in the levels of Fis1 and Drp1 proteins without affecting mitofusin 2. A decrease in the level of Drp1 phosphorylated at its amino acid residue 637 was also detected (S2A–S2E Fig). This prompted us to test whether inhibiting Drp1 function by a dominant-negative mutant K38A-Drp1 could reverse FUS-induced mitochondrial fragmentation in cultured neurons. As shown in S3A–S3C Fig, we co-transfected mitoRed and GFP control, Wt-FUS-GFP or P525L-FUS-GFP together with either the vector control, wild type Drp1-Flag (WtDrp1) or K38A mutant Drp1-Flag (K38A-Drp1). Expression of Wt-Drp1 moderately increased the percentage of neurons with fragmented mitochondria as compared with the control group, whereas expression of K38A-Drp1 mutant decreased the percentage of cells containing fragmented mitochondria, especially in the neurons expressing the P525L-mutant FUS (S3A–S3C Fig). Moreover, the percentage of neurons showing condensed nuclei was also reduced by the expression of K38A-Drp1 in those expressing the P525L-mutant FUS. It prompted us to test if inhibiting Drp1 in FUS transgenic flies would rescue the neurodegenerative phenotypes. However, expression of Drp1-specific RNAi or a dominant-negative K38A-Drp1mutantdid not lead to a significant rescue of neurodegeneration phenotypes in the FUS transgenic flies expressing Wt- or P525L-FUS protein (S3D and S3E Fig). It has been reported that a dominant- negative mutation in Drp1 is associated with a severe neurodevelopmental syndrome in a patient and down-regulation of Drp1 leads to developmental defects in flies [51,52]. It is possible that simply suppressing Drp1 activity is not sufficient to block the FUS-induced neurotoxic signal(s).

### FUS is associated with mitochondria, with the ALS-associated P525L-mutant showing increased FUS mitochondrial localization

**Fig 3:**
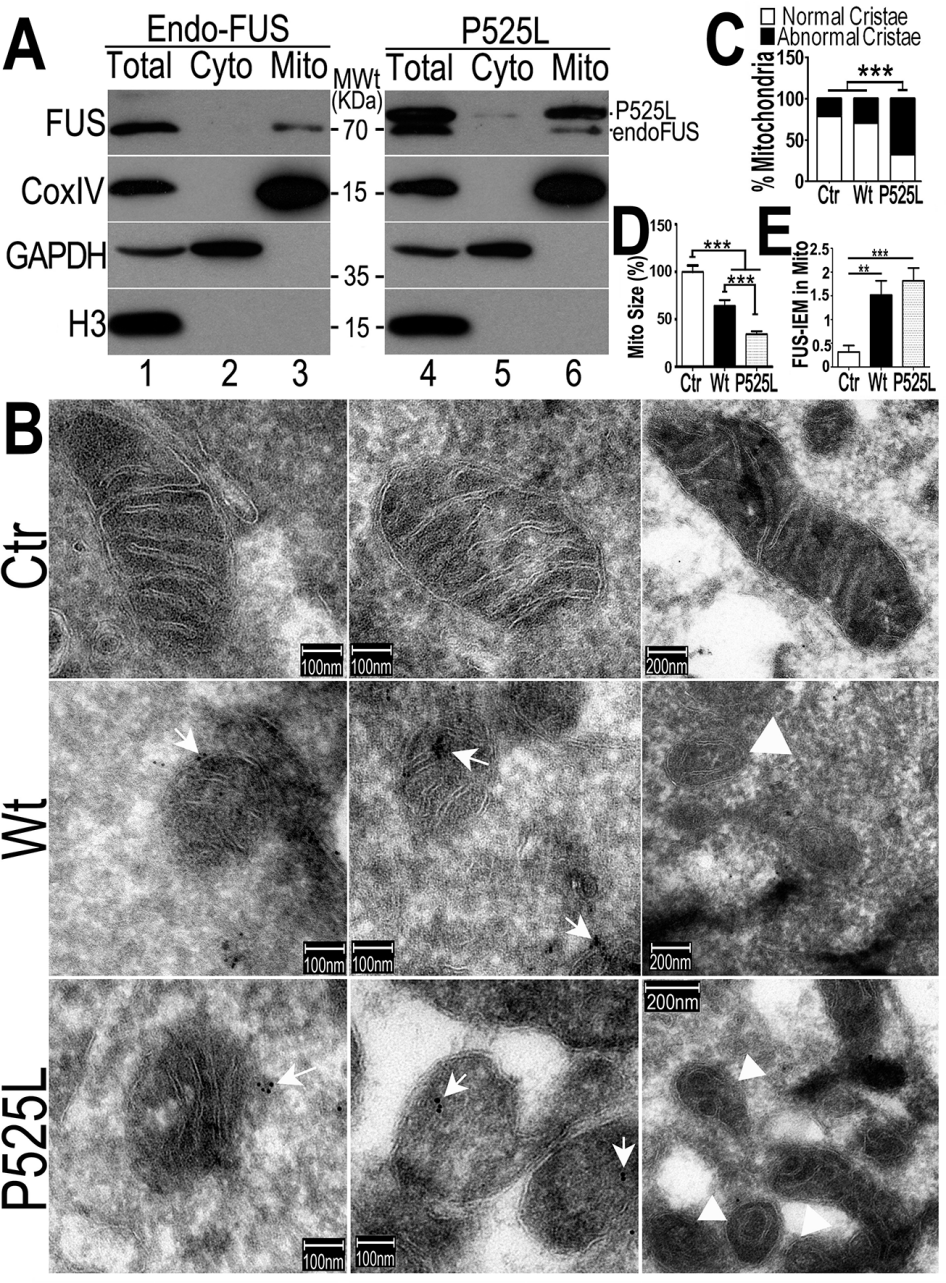
The FUS protein is associated with mitochondria. (A)Highly purified mitochondria were prepared from the control or P525L-FUS-expressing stable HEK cell lines. The mitochondrial purity was confirmed by the detection of mitochondrial CoxIV and the absence of the cytoplasmic proteins such as GAPDH or nuclear protein Histone H3. The endogenous FUS or P525L-mutant FUS localized to mitochondria; and the P525L-mutant FUS showed increased levels of mitochondrial localization (lane 6), as compared with the endogenous FUS in mitochondria (lane 3). (B-D) IEM images of the control or FUS-expressing stable HEK cell lines show reduced mitochondrial sizes in cells overexpressing FUS. Arrows, FUS-immunostaining signals associated with mitochondria labeled with 10-nm immuno-gold particles; arrowheads, mitochondria showed damaged cristae with “onion-like” structure. Mitochondrial cristae in P525L-mutant FUS expressing cells were significantly more frequently disrupted than the control and Wt-FUS groups, with quantification shown in panel C. More than 50 mitochondria were quantified in each group, analyzed using Chi-square test (***:p<0.0001).(D) Quantification of mitochondrial size using Image J. Mitochondria in Wt- or P525L-mutant FUS expressing cells were significantly smaller than the control group. At least 50 mitochondria were quantified in each group, analyzed using one-way ANOVA with Bonferronipost test (***: p<0.0001). (E) Mitochondrion-associated FUS immunostaining signals were significantly increased in Wt or P525L FUS expressing cells as compared with the Ctr. At least 60 mitochondria were quantified in each group, analyzed using one-way ANOVA with Bonferronipost test (**: p<0.01; ***: p<0.0001).

To examine the relationship between FUS and mitochondria, we purified mitochondria from the HEK293 cells expressing the vector control or the ALS-associatedP525L-mutant FUS following published protocols [53,54] (Fig 3). Western blotting experiments demonstrate that our mitochondrial preparations were highly enriched in mitochondrial proteins (such as CoxIV) but devoid of either cytoplasmic proteins (such as GAPDH) or nuclear proteins (e.g., Histone H3) (Fig 3A). The endogenous FUS protein was consistently detected in these highly purified mitochondrial preparations (lane3 in Fig 3A). Although the level of P525L-mutant FUS protein detected was higher than the endogenous Wt-FUS protein in both the purified mitochondria and cytosol (see Fig 3A, the upper band was the P525L-mutant FUS; the lower band, the endogenous FUS), the endogenous FUS protein was clearly detected in the purified mitochondria (Fig 3A, lane 3 and lane 6). These data demonstrate that both the endogenous wild-type and transfected ALS-mutant FUS are translocated to mitochondria.

To confirm that the FUS protein is indeed closely associated with mitochondria, we performed immuno-electron microscopy (IEM) using the specific anti-FUS antibody. In the control HEK293 cells, a fraction of FUS-IEM staining signals were associated with mitochondria, whereas in cells overexpressing the Wt- or P525L-mutant FUS, many mitochondria were decorated with immuno-gold particles conjugated to the specific anti-FUS antibody (arrows in Fig 3B and 3E). It should be noted that the anti-FUS antibody was specific, because pre-absorption of the antibody using the purified FUS antigen essentially eliminated the immunoreactive signals in Western blotting (WB) and IEM (see S1A–S1D Fig). Consistently, down-regulation of FUS by specific siRNA (siFUS) or genetic deletion of the FUS gene reduced or eliminated the WB signals (see S1C and S1D Fig, respectively).Quantification of IEM images shows that mitochondria in cells expressing either Wt- or P525L-mutant FUS were smaller with severely damaged cristae, as compared with those in the control group (Fig 3B–3D). “Onion-like” mitochondria with multi-layered structure and damaged cristae were frequently detected in cells expressingP525L-mutant FUS (marked by arrowheads in Fig 3B and 3C), but not in the control cells. Consistent with the data described previously, mitochondrial size was significantly decreased in cells expressing either Wt- or P525L-mutant FUS, with the P525L-mutant showing more pronounced mitochondrial defects (Fig 3D). These data support the notion that increased FUS expression leads to mitochondrial damage. Together, our results demonstrate that increased FUS expression, especially that of the cytoplasm-localized ALS-associated P525L-mutant FUS protein, promotes association of FUS with mitochondria and induces mitochondrial damage.

### Expression of Wt- or ALS-associated mutant FUS reduces the mitochondrial membrane potential and increases the production of mitochondrial reactive oxygen species

**Fig 4:**
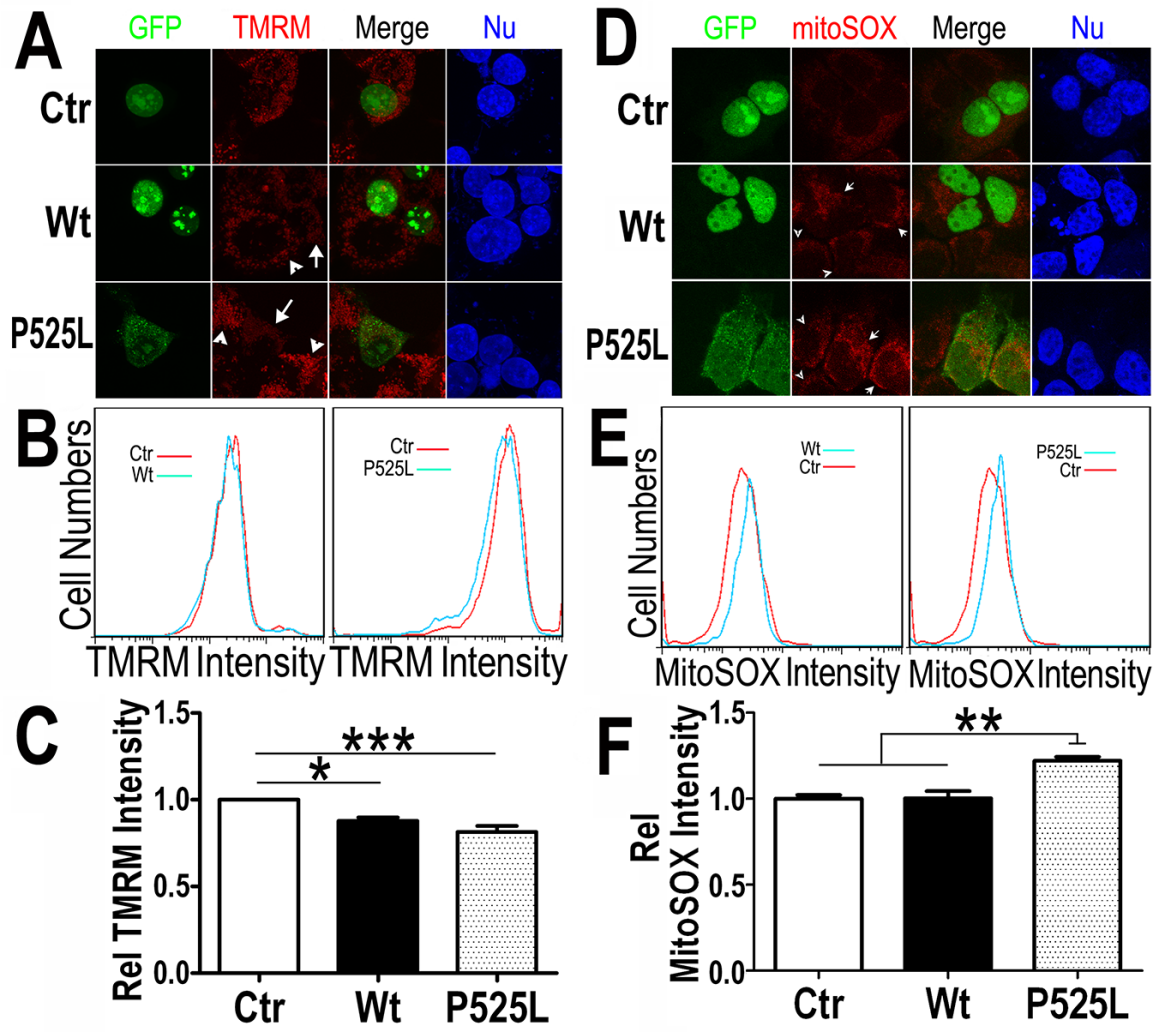
Increased expression of Wt- or P525L-mutant FUS induced a decrease in mitochondrial membrane potential and an increase in production of mitochondrial superoxide. (A) Confocal images of live TMRM-stained HEK293 cells following transfection with corresponding plasmids: the GFP control, Wt- or P525L-mutant FUS tagged with GFP. All images were acquired in Z-stacks and projected as single images. Cells were stained with TMRM (a mitochondrial membrane potentiometric dye) and Hoechst 33342 before imaging.(B, C)FACS analyses of GFP-positive cells from experiments in panel A with quantification of TMRM signal intensity. Cells expressing either Wt- or P525L-mutant FUS showed decreased TMRM intensity as compared with the control group. The data were analyzed using one-way ANOVA (representing 4 independent experiments; *: p< 0.05, ***: p< 0.001). (D) Confocal images of mitoSox Red-stained HEK293 cells 24-hrs following transfection as described in panel A and Hoechst 33342 staining. (E, F) FACS analyses and quantification of mitoSOX-Red stained cells shown in panel D. Cells expressing P525L-mutant FUS showed significantly increased mitoSOX staining signals as compared with cells expressing the control or Wt-FUS protein. Data from 3 independent experiments were analyzed using one-way ANOVA (**:p< 0.01).

A common sign of mitochondrial damage is the change in mitochondrial membrane potential, leading to mitochondrial depolarization. We tested if FUS expression affects mitochondrial membrane potential. Tetramethylrhodamine methyl ester (TMRM), a fluorescent dye labeling mitochondria in a membrane potential-dependent manner [55] was used to stain HEK293 cells expressing either the GFP vector control or Wt- or P525L-mutantFUS tagged with GFP. In cells expressing the control, the TMRM signals were detected at a similar level as in the neighboring non-transfected cells (Fig 4A). However, the TMRM staining intensity in cells expressing Wt- or P525L-mutant FUS (marked by the white arrows) was reduced as compared with their neighboring non-transfected cells (marked by arrowheads). The control and Wt- or P525L-mutant FUS expressing cells were then analyzed by fluorescence activated cell sorting (FACS) to quantify TMRM florescence intensity in GFP-positive cells. Consistently, TMRM intensity was significantly lower in cells expressing either Wt- or P525L-mutant FUS as compared with the GFP vector-control group, with the P525L-mutant group showing more pronounced effect (Fig 4B and 4C). This indicates that FUS expression, in particular that of P525L-mutant, reduces mitochondrial membrane potential and causes mitochondrial damage.

Next, we measured the mitochondrial production of the reactive oxygen species (ROS) in these cells. Using a specific mitochondrial superoxide indicator, mitoSOX, we compared cells expressing either Wt- or P525L-mutant FUS. Both confocal images and FACS analyses showed that ROS production was increased in the P525L-mutant FUS expressing cells (Fig 4D–4F). It has been reported that ROS could activate Drp1 and induce mitochondrial fragmentation [56], consistent with our findings of FUS-induced Drp1 activation and mitochondrial damage.

### Increased FUS protein levels and profound mitochondrial damage are detected in FTLD-FUS brain samples

**Fig 5:**
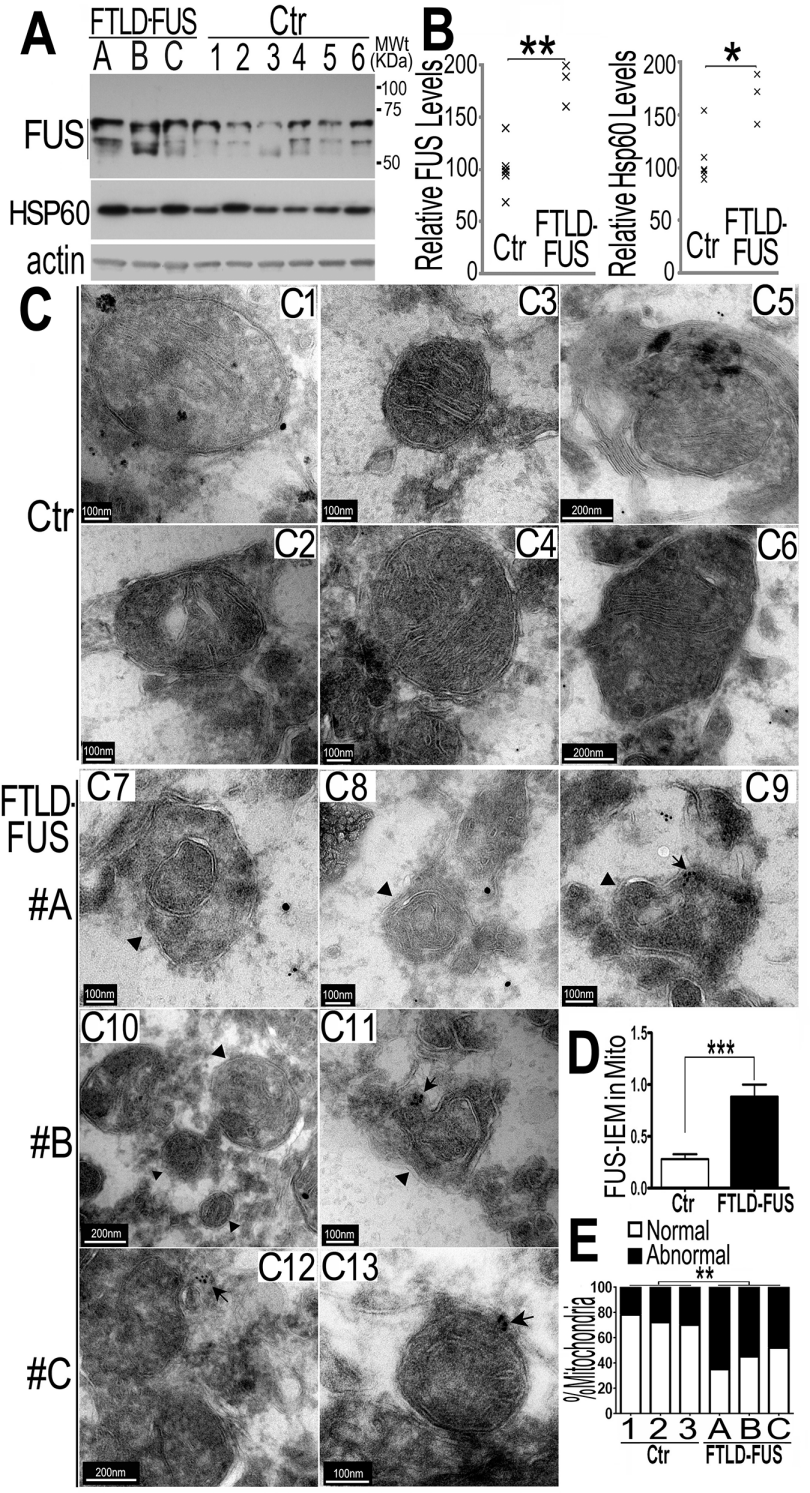
Increased FUS protein levels and mitochondrial damage detected in 3 independent FTLD-FUS brain samples. (A, B)Fronto-cortical tissues of postmortem brain samples from three patients diagnosed with FTLD-FUS (#A, B and C) or 6 control cases (Ctr; #C1-#C6) were lysed in 1%SDS-containing hot lysis buffer (see Methods) and used for Western blotting with anti-FUS or HSP60 antibodies. Beta-actin was used as an internal control. In panel B is quantification of data in panel A, representing 3 independent samples (*: P<0.05;**: p<0.01; ANOVA with student t test). (C) IEM of FTLD-FUS and control brain samples. Cryosections of corresponding brain samples were immuno-labeled with specific anti-FUS antibody and secondary anti-murine IgG conjugated to 10-nm gold particles. Mitochondria were labeled with rabbit-anti-Tomm20 and anti-rabbit IgG conjugated to 25-nm gold particles. Arrows mark FUS-immunostaining signals labeled with 10-nm immuno-gold particles; arrowheads, mitochondria showing damaged cristae.(D) Mitochondrion-associated FUS immunostaining signals were significantly increased in FTLD-FUS cases as compared with the controls(***: p<0.0001). (E) Mitochondrial cristae showed significant damage in FTLD-FUS samples as compared with the control samples. At least 30 mitochondria were quantified in each group and analyzed using Chi-square test (**:p<0.01).Data represent three independent experiments.

To characterize molecular and cellular damages in patients with FUS-proteinopathies, we collected de-identified post-mortem tissue samples from the Cognitive Neurology & Alzheimer's Disease Center at Northwestern University. After initial testing to exclude cases with non-specific protein degradation, three FUS-proteinopathies brains together with six control samples were identified suitable for biochemical studies and electron microscopy. In all three FUS-proteinopathies cases examined, no FUS mutations were identified (see S1 Table) and the pathological diagnosis was FTLD-FUS with prominent FUS-positive inclusion bodies detected in the brain tissues. Using the specific anti-FUS antibody, we examined FUS protein levels in these FTLD-FUS brain samples together with six control samples. Remarkably, all three FTLD-FUS samples showed increased FUS protein levels as compared to the controls (Fig 5A and 5B). This is consistent with a previous report that the brain samples from patients affected by atypical FTLD with FUS pathology (aFTLD-FUS) showed increased total FUS levels, as compared with the controls or FTLD-TDP-43 samples [10]. It should be noted that 2–3 bands were detected in the human brain samples, with approximate molecular weight of 53-70kD, which is consistent with previously published Western blotting data on FUS proteinopathies patient tissue samples [57,58]. It is possible that FUS may undergo proteolytic cleavage, and the molecular nature of such cleavage remains to be determined by future studies. Because our mass-spectrometry analyses showed mitochondrial HSP60 as a FUS-interacting protein, we also examined HSP60 protein in these brain samples and found that two FTLD-FUS cases (A and C) exhibited elevated HSP60 protein level (Fig 5A and 5B). It is not surprising to see that not all FTLD-FUS samples exhibited increased HSP60 expression. Considering the multi-level regulation of FUS gene expression and the diverse genetic background in different individuals, it is conceivable that different mechanisms contribute to the development of FUS-proteinopathies and that HSP60 is not the only modulator of FUS induced neurotoxicity among different FTLD-FUS patients.

We performed IEM of these samples using the FUS-specific antibody and the secondary antibody conjugated to gold particles. In the control samples, most mitochondria appeared healthy with well-organized cristae as packed-stacks of membrane sheets (see panels C1-C6 in Fig 5C) and with only a few FUS-immunostaining signals detected in the vicinity of the mitochondria. However, in all three FTLD-FUS brain samples, FUS-immuno-positive signals were frequently detected in close association with mitochondria (arrows in panels C9-C13 in Fig 5C and 5D). Consistently, mitochondria in these FTLD-FUS cases showed a marked loss or disruption of cristae with frequent detection of “onion-like” deformed shape (arrowheads in panels C7-C13 in Fig 5C). Quantification of the EM data indicates that all three FTLD-FUS cases showed increased FUS-immunostaining signals in mitochondria and increased mitochondrial damage (Fig 5D and 5E). These observations indicate that mitochondrial impairment, accompanied by increased FUS expression, represents a prominent neuropathological feature in FTLD-FUS patients.

### HSP60 interacts with FUS and mediates FUS mitochondrial localization

**Fig 6:**
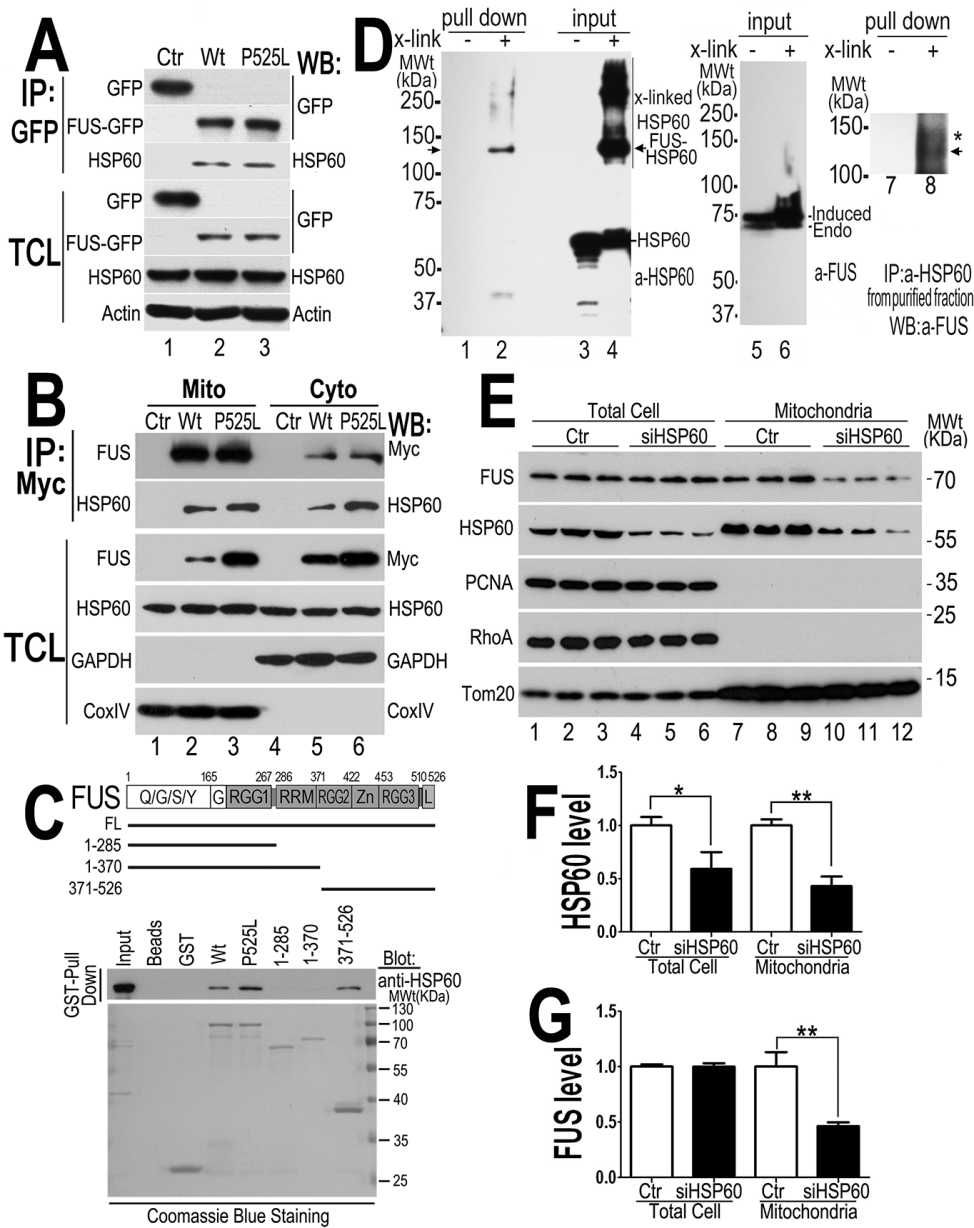
HSP60 interacts with FUS, mediating FUS mitochondrial localization. (A) FUS-HSP60 interaction was detected by co-immunoprecipitation assay. Western blotting (WB) was performed using corresponding specific antibodies following immunoprecipitation of cell lysates with anti-GFP.(B)FUS-HSP60 interaction was detected by co-immunoprecipitation assay in both mitochondrial and cytosolic fractions. WB was performed using corresponding specific antibodies following immunoprecipitation of mitochondrial or cytosolic fractions with an anti-Myc monoclonal antibody. It should be noted that the protein concentrations of purified mitochondrial fraction relative to the cytosolic fraction is 10 to 1 and that approximately 5% of total FUS protein is localized to mitochondria.(C) GST pull-down experiments using purified FUS and HSP60 proteins indicate that the full-length (FL) Wt- or P525L-mutant FUS or the carboxyl terminal fragment (aa371-526) of FUS protein interacts with HSP60. GST pull-down proteins were analyzed by WB using the anti-HSP60 antibody. The lines below the FUS protein domain diagram depict different truncation mutants of FUS as GST-tagged proteins, with the corresponding amino acid residues shown. The lower panel of the gel image shows Coommassie blue staining of corresponding purified GST-FUS fusion proteins used. (D) Detection of direct FUS-HSP60 interaction by cross-linking coupled immunoprecipitation in live cells expressing the P525L-mutant FUS as a 6XHis-Myc tagged protein. The 130kDa cross-linked FUS-HSP60 species was detected by WB using the anti-HSP60 antibody in the pull-down proteins (“pull down”) following tandem-affinity purification using His-resins and anti-Myc beads (lane 2) or using anti-FUS in the “pull down” proteins following further immunoaffinity purification by anti-HSP60 (lane 8). Briefly, following 16-hour tetracycline induction of FUS expression, the control orP525L-mutant FUS expressing cells were treated with a cross-linking agent, formaldehyde (1%) for 10 min. FUS proteins were tandem-affinity purified using His-resin and anti-myc antibody and then analyzed by WB. A band was detected by anti-HSP60 antibody at 130-kDa in the pull-down fraction as marked by the arrow, whereas multiple bands including 60- and 130-kDa were detected in the input cross-linked cell lysates (input). HSP60 and the tagged FUS were detected at a size of 60 and 70 kDa, respectively, consistent with the prediction that the HSP60 containing 130kDa band consists of HSP60 and 6XHis-Myc tagged FUS. (E) Down-regulating HSP60 leads to a reduction in FUS mitochondrial localization. HEK293 cells were transfected with the control or HSP60-specific siRNAs and harvested for mitochondrial purification 72-hr post-transfection. The mitochondrial purity was confirmed by the enrichment of mitochondrial TOM20 and the absence of cytoplasmic protein such as RhoA or nuclear protein PCNA. Triplicates of the experiments are shown. (F) Quantification of the HSP60 levels in the total cell extracts and in the mitochondrial fractions. (G) Quantification of FUS levels in total cell extracts and the mitochondrial fractions. All data represent 3 independent experiments with statistical analyses using one-way ANOVA with Bonferronipost-test (*: p<0.05; **: p<0.01).

HSP60, an ATP-dependent mitochondrial chaperone protein associated with neurodegeneration [30] was one of the FUS-interacting mitochondrial proteins identified in our immunoaffinity-coupled mass-spectrometry experiments. We confirmed the FUS-HSP60 interaction in a co-immunoprecipitation (co-IP) assay using HEK293 cells transfected with the Wt- or P525L-mutant FUS tagged with GFP or the GFP vector control. Immunoprecipitation to pull-down FUS followed by Western blotting using the HSP60-specific antibody revealed that both Wt- and P525L-mutant FUS interacted with HSP60 (Fig 6A). To examine whether FUS interacts with HSP60 in the cytosol or in mitochondria, we prepared mitochondrial and cytosolic fractions from HEK293 cells expressing the control vector, Wt- or P525L-mutant FUS tagged with 6xMyc tag to perform co-IP assay. Western blotting analyses of the immunoprecipitated protein revealed that Wt- or P525L-mutant FUS interacted with HSP60 in the mitochondria as well as in the cytosol (Fig 6B).To test whether FUS directly interacted with HSP60, we performed a cross-linking immunoprecipitation assay (Fig 6D, S4B Fig). Following the treatment with formaldehyde (a cross-linking reagent) of the live cells stably expressing 6xMyc-His tagged FUS, FUS protein was purified by pulling-down with Ni-NTA resin. A 130 kDa species was detected by anti-HSP60 antibody, representing FUS-HSP60 cross-linked product, because Myc-His tagged FUS and HSP60 were detected as 70 and 60kDa proteins, respectively (marked by the arrow, lane 2 and lane 8 in Fig 6D). Because live cells were treated with the cross-linking reagent immediately before immunoprecipitation in this assay, our data support the notion that FUS directly interacts with HSP60 in cells. In addition, protein purification was performed under the denaturing condition in the presence of guanidine-HCl using Ni-NTA resin, thus it is unlikely that the 130kDa cross-linked product was a result indirect interaction of HSP60 with FUS. To further demonstrate that FUS directly interacts with HSP60 and to map the region in FUS responsible for FUS-HSP60 interaction, we performed glutathione S-transferase (GST) pull-down experiments using purified GST-tagged proteins of either Wt- or P525L-mutant or fragments of the Wt-FUS protein and His-tagged HSP60 protein (see Fig 6C). Although the interaction was not detectable when the N-terminal fragments containing 285 or 370 amino acid residues (aa), the full-length Wt- or P525L-mutant or the carboxyl terminal fragment containing aa371-526 clearly interacted with the purified HSP60 protein (Fig 6C). To test if the FUS-HSP60 interaction is RNA-dependent, we performed GST pull-down experiments in the presence of RNaseA. RNase A treatment did not affect the interaction between FUS and HSP60, although there was partial degradation of FUS and HSP60 proteins following RNaseA treatment (S4A Fig). Together, these data indicate that Wt- or P525L-FUS directly interacts with HSP60 in cells and *in vitro*.

To test whether HSP60 mediates FUS mitochondrial localization, we down-regulated HSP60 expression using specific siRNA in HEK293 cells and then examined the level of mitochondrion-localized FUS (the endogenous wild-type FUS) using purified mitochondria followed by Western blotting. Although the total FUS levels were not altered, the mitochondrial FUS level was significantly decreased in the HSP60-siRNA transfected cells, as compared with the control-siRNA group (Fig 6E–6G). Furthermore, analyses of the nuclear and cytosolic fractions revealed that the nuclear FUS level was increased in the HSP60-siRNA transfected cells, as compared with the control-siRNA group (S4C–S4E Fig). We also performed siRNA knock-down experiments in the stable cells expressing the P525L-mutant FUS. Similarly, reducing HSP60 expression in these cells also decreased mitochondrial localization of both the P525L-mutant and the endogenous Wt-FUS proteins (S4F–S4H Fig). These results suggest that HSP60 may play an important role in mediating FUS translocation to mitochondria.

### Reducing HSP60 expression rescues neurodegenerative phenotypes in FUS transgenic flies

**Fig 7:**
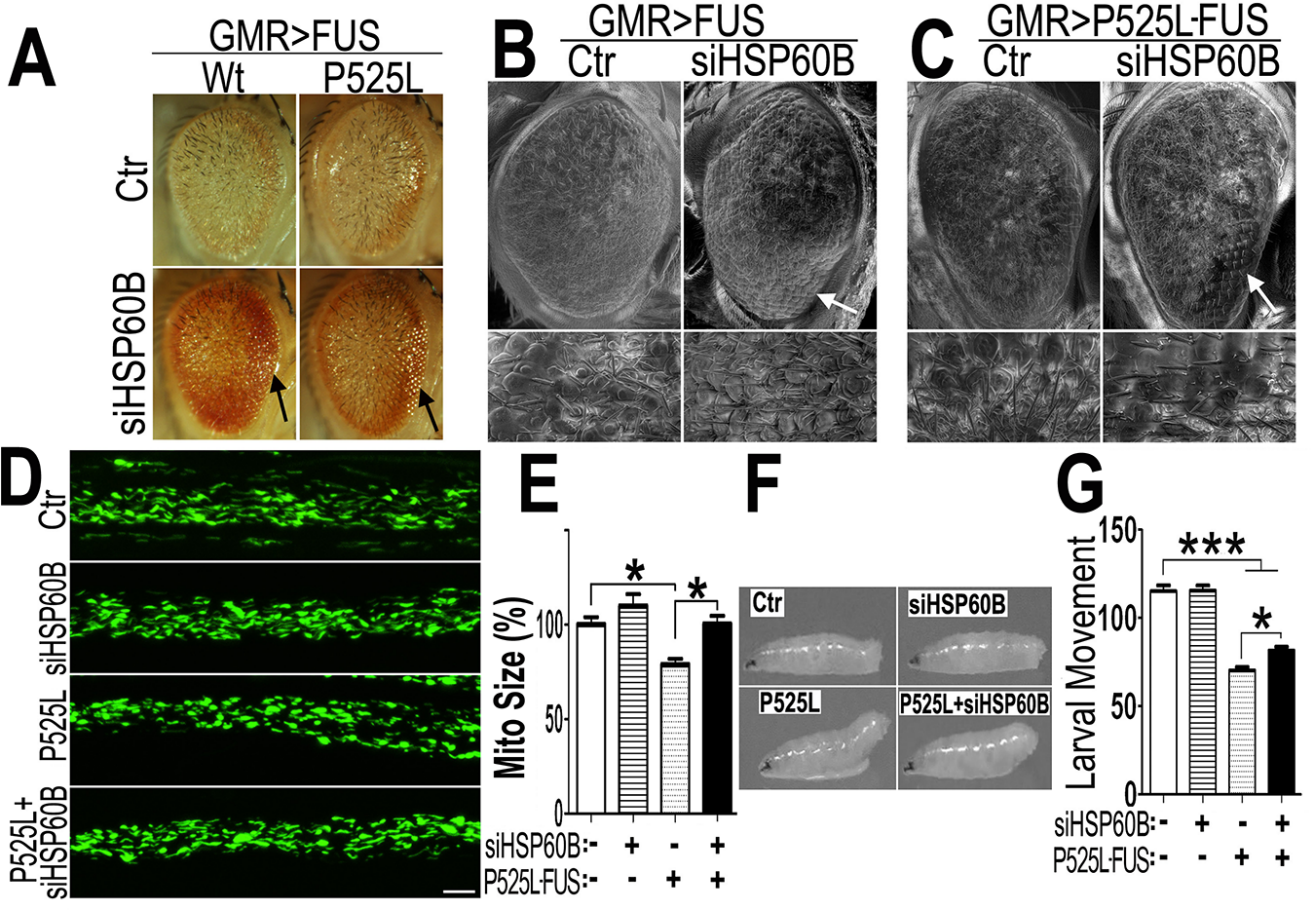
Down-regulating fly HSP60B expression partially rescues neurodegeneration phenotypes in FUS transgenic flies. (A)FUS genetically interacts with HSP60B. Co-expressing siHSP60B with FUS in transgenic flies significantly rescued FUS-induced retinal defects as compared with the control. (B, C)Scanning electron microscopic images of fly eyes expressing FUS together with the control siRNA or siHSP60B, with the lower panels showing higher magnification images of the corresponding groups. Flies co-expressing siHSP60B and FUS showed more well-organized bristles and reduced ommatidial fusion as compared with FUS transgenic flies expressing the control siRNA. Fly genotypes: Ctr: GMR-Gal4/UAS-Wt-FUS-RFP or GMR-Gal4/UAS-P525L-FUS-RFP; siHSP60B: GMR-Gal4/UAS-Wt-FUS-RFP/UAS-siHSP60B or GMR-Gal4/UAS-P525L-FUS-RFP/UAS-siHSP60B. (D)Confocal microscopic images of mitochondria in motor neuron axons in the larval A3 abdominal segments(Z-stacks projected into single images). Fly genotypes, Ctr: D42-Gal4/UAS-mitoGFP/UAS-RFP; siHSP60B: D42-Gal4/UAS-mitoGFP/UAS-siHSP60B; P525L: D42-Gal4/UAS-mitoGFP/UAS-P525L-FUS-RFP; P525L-FUS+siHSP60B: D42-Gal4/UAS-mitoGFP/UAS-P525L-FUS-RFP/UAS-siHSP60B. (E) RNAi mediated down-regulation of HSP60B in P525L-mutant FUS expressing flies rescued mitochondrial fragmentation phenotype, as shown by quantification of mitochondrial lengths in MN axons. The 3rd instar larvae expressing P525L-mutant FUS showed significantly smaller mitochondria as compared with the control. Ten larvae were measured in each group. (F, G)Knocking-down HSP60B in flies expressing P525L-mutant FUS in MNs significantly rescued their larval locomotive defects. The tail paralysis phenotype was examined in third instar larvae, as measured by the loss of ability for larvae to anchor their tails on the agar surface when crawling {as previously published [17]}. Fly genotypes, Ctr: OK371-Gal4/UAS-RFP; siHSP60B: OK371-Gal4/UAS-siHSP60B; P525L: OK371-Gal4/UAS-P525L-FUS-RFP; P525L-FUS+siHSP60B: OK371-Gal4/UAS-P525L-FUS-RFP/UAS-siHSP60B. All data were analyzed using one-way ANOVA with Bonferroni post-test (*: p<0.05; ***: p<0.001).

To examine HSP60-FUS interaction *in vivo*, we tested whether FUS genetically interacted with HSP60 in flies by knocking-down Drosophila homologs of human HSP60, including HSP60A, B, C or D in FUS transgenic flies using RNA interference (RNAi) (supplementary S5 and S6 Figs). Fly eyes expressing Wt- or P525L-mutant FUS exhibited rough surface and reduced pigmentation. Scanning electron microcopy (SEM) revealed ommatidial loss, ommatidial fusion, ectopic bristle formation and disrupted ommatidial organization, as reported previously [17]. Down-regulating expression of HSP60A, HSP60B orHSP60C in photoreceptors suppressed the FUS-induced retinal degeneration phenotype to various extents, with siHSP60B showing the most robust effect, although knocking-down HSP60 did not affect the expression level of FUS (see S5A and S5B Fig). Therefore, we chose siHSP60B for subsequent experiments. Knocking-down HSP60B in FUS-expressing flies partially restored the eye morphology, rescuing FUS-induced photoreceptor degeneration, although siHSP60B by itself in the control flies did not show any effects (Fig 7A; S7 Fig). Scanning EM revealed that down-regulating HSP60B in fly eyes expressing Wt- or P525L-mutant FUS partially reversed FUS-induced ommatidial loss or fusion, restoring ommatidial pattern and bristle organization, especially in the peripheral region of the eye (Fig 7B and 7C). The effect of siHSP60 in rescuing FUS-induced neurodegeneration was specific because a number of fly lines tested that expressed siRNAs against other genes did not show any effect (see S3D Fig). When mitochondria were examined in the motor neuron axons, reducing HSP60B expression by RNAi alone in the control flies did not affect mitochondrial size (Fig 7D and 7E). In contrast, down-regulating HSP60B significantly increased the mitochondrial size in flies expressing P525L-mutant FUS in motor neurons (Fig 7D and 7E), thus rescuing the effect of P525L-mutant FUS induced mitochondrial fragmentation. Importantly, HSP60B down-regulation in fly MNs significantly rescued the locomotive defects in the P525L-mutant FUS expressing fly larvae, with mitigated tail-paralysis phenotype in these siHSP60B/P525L-FUS expressing animals (Fig 7F and 7G). These results indicate that FUS genetically interacts with HSP60, suggesting a role of HSP60 in FUS-induced neurotoxicity.

**Fig 8:**
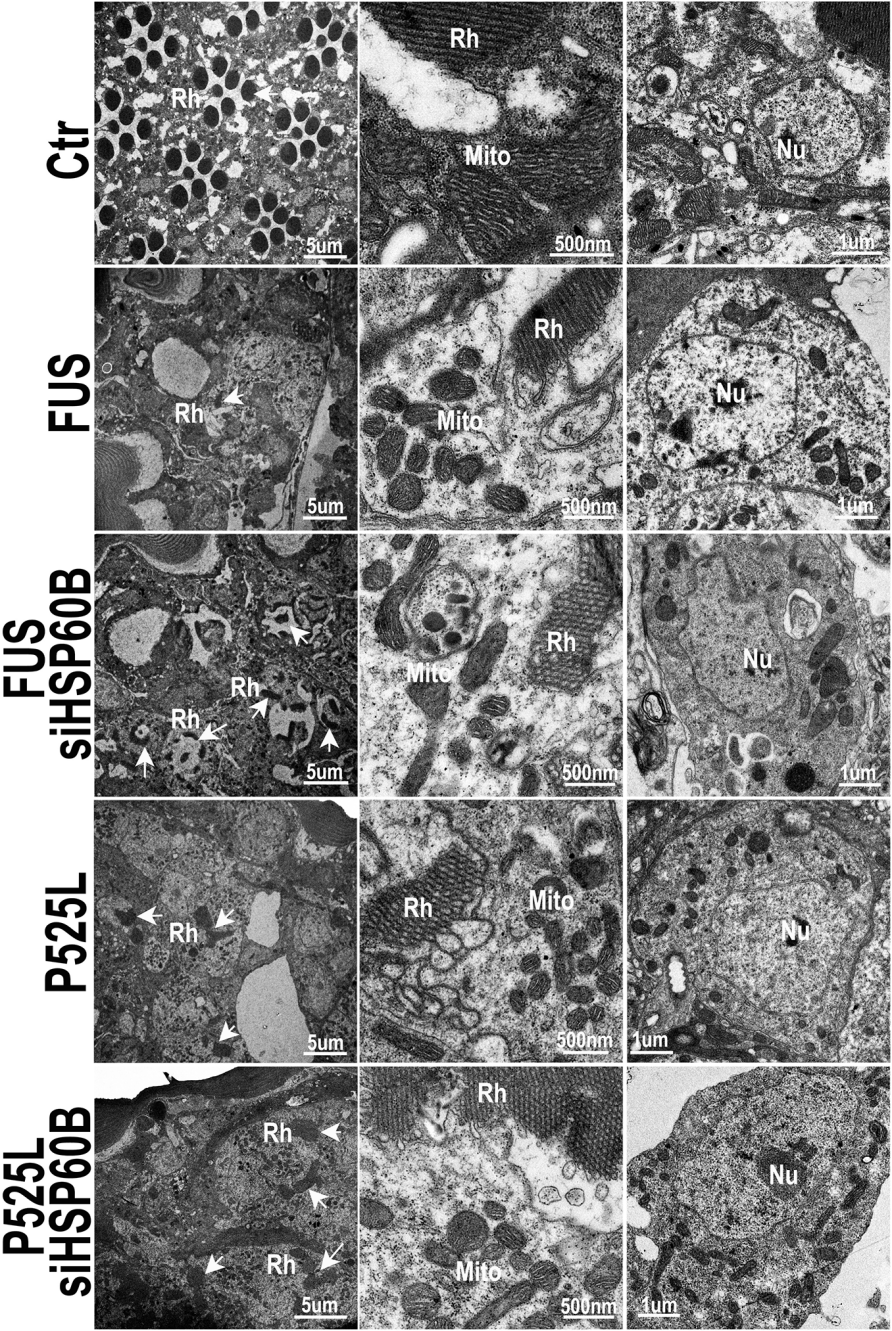
Down-regulating HSP60B in fly photoreceptors partially rescues the retinal degeneration phenotype of FUS transgenic mice as shown by transmission electron microscopy (TEM). Electron microscopic images of fly retinas at day 3 are shown, together with mitochondria and nuclei (at higher magnifications). Rh: rhabdomere (marked by arrows); Mito: mitochondria; Nu: nuclei. The retinas in the control flies display normal rhabdomere organization with seven photoreceptor cells and healthy mitochondria at day 3, but the flies expressing either Wt- or P525L-mutant FUS show disrupted rhabdomere organization and smaller mitochondria. Knocking-down HSP60B expression by specific siRNA partially rescued these defects in flies expressing Wt- or P525L-mutant FUS.

Then, we used transmission EM (TEM) to examine the fly retinal structure. Retinas displayed normal differentiation of seven rhabdomeres at day 3, whereas the flies expressing Wt- or P525L-mutant FUS exhibited defective rhabdomeres (marked as “Rh” in Fig 8). Although knocking-down HSP60B in files expressing Wt- or P525L-mutant FUS did not completely restore the rhabdomeres to its normal morphology, it improved rhabdomere formation in the siHSP60B-expressingflies (as marked by arrows in Fig 8)(S8D Fig). As compared with the healthy mitochondria in the control flies, the mitochondria in the flies expressing Wt- or P525L-mutant FUS were smaller (S8E Fig). Down-regulating HSP60B expression in these FUS transgenic flies significantly increased the mitochondrial size (S8E Fig). It was noticed that at day 3 the nuclei of photoreceptor cells in the fly eyes expressing either Wt- or P525L-mutant FUS appeared swollen with nearby mitochondria severely damaged, although the cells remained intact. This phenomenon was not detected in the control flies (Fig 8, S8B and S8C Fig). By day 15, a significant photoreceptor loss was observed in flies expressing Wt- or P525L-mutant FUS as compared with the control flies, or with the FUS transgenic flies at day 3 (Fig 8, S8A Fig). These results support the hypothesis that mitochondrial damage may be an early event in FUS proteinopathies and that the mitochondrial damage induced by FUS expression may precede photoreceptor neuronal death.

## Discussion

Originally identified as a gene involved in chromosomal translocation in liposarcoma [59], the human FUS gene encodes a RNA/DNA binding protein involved in multiple cellular processes [60,61]. Although it has been reported that FUS shuttles between the nucleus and cytoplasm [62] and that FUS has many nuclear activities, the cytoplasmic function of FUS is far less clear. For the first time, our study reveals that the FUS can be localized to mitochondria and increased FUS mitochondrial localization is toxic to neurons, contributing to neurodegeneration. While the physiological role of FUS in regulating mitochondrial biology remains to be elucidated by future studies, our data provide new information about the cytoplasmic platform on which FUS is likely to play an active role.

The vast majority of mitochondrial proteins are encoded by the nuclear genome and synthesized in the cytoplasm, then transported into mitochondria via different mechanisms [63]. Under physiological conditions, FUS possibly plays a functional role in mitochondria. A recent report showed that knocking down of FUS by RNAi affected the expression of a subset of mitochondrion-associated genes [64]. Although the human FUS protein shows a very low probability (<0.0001) in computational prediction of mitochondrial import using a published algorithm [65], our data, ranging from biochemical purification of mitochondria, immunoEM detection of the endogenous FUS to *in vivo* assays in transgenic flies provide clear evidence that FUS is associated with mitochondria. Previous studies have shown that some mitochondrial proteins are chaperoned by heat shock proteins [66]. The mitochondrial chaperonin HSP60, together with HSP70 and HSP10, are important for protein import into mitochondria [30,32,33,34,35,36]. Although it has been reported that HSP60 may promote mitochondrial localization of amyloid-beta peptide and mitochondrial impairment in Alzheimer disease [67], the role of HSP60 in other neurodegenerative diseases remains unclear. Mutations in HSP60 were reported to be associated with spastic paraplegia type 13 (SPG13), a late-onset autosomal-dominant neurodegenerative disease [37,38]. Recently, it was reported that a heterozygous knock-out HSP60 mouse model could recapitulate the features of human disease (SPG13) and increase mitochondrial ROS [68]. Here, we demonstrate that FUS interacts with HSP60 by mass-spectrometry analyses of FUS interacting proteins, co-immunoprecipitation, GST pull-down and cross-linking experiments. Importantly, down-regulation of HSP60 expression decreased mitochondrial FUS levels and partially rescued FUS-induced phenotypes, including mitochondrial fragmentation, neurodegeneration and locomotive deficits. Drosophila has four HSP60 homologs. In our experiments, knocking down expression of each HSP60 homolog by their RNAi led to a partial rescue of the neurodegenerative phenotypes in FUS transgenic flies. It is likely that different HSP60 homologs or HSP60-like genes may compensate for each other *in vivo*.

Our data support the hypothesis that HSP60 promotes or mediates FUS translocation from the nucleus to mitochondria. HSP60 has been reported to play a dual role in cell death, as either an anti- or a pro- apoptosis factor [69]. On one hand, HSP60 together with HSP70 may play a protective role in mitochondrial unfolded protein response [33]. HSP60 may form a pro-survival complex with Bcl-2, an anti-apoptotic mediator; and disruption of this complex formation by nutrient deprivation results in cell death [70]. On the other hand, HSP60 has been reported to facilitate pre-caspase-3 maturation [71] or induce nitric oxide production by microglia, leading to neurotoxicity [72]. Our results indicate that HSP60 interacts with FUS to promote mitochondrial damage and cell death. Future studies are necessary to determine whether modulating the HSP60-FUS interaction may alter the activity of HSP60 in regulating neuronal survival. For example, it is possible that the interaction of FUS with HSP60 might affect the ability of HSP60 to form complex with Bcl-2, thereby inhibiting Bcl-2 pro-survival function and resulting in cell death. Our findings provide a new direction for further investigating the multi-facet role of HSP60 in neurodegenerative disorders.

There are four HSP60 homolog genes in drosophila, named as HSP60A, HSP60B, HSP60C, HSP60D [73,74]. The predicted phylogenetic relationship between these four genes and human HSP60 is shown in S6 Fig. HSP60A is a constitutively expressed chaperonin in drosophila cells and essential for embryogenesis. Mutations in HSP60A lead to embryonic lethal phenotype in Drosophila [75]. HSP60B shares extensive homology with other drosophila HSP60 proteins and is required for spermatid individualization process. Mutations in HSP60B result in male sterility [76]. HSP60C is required for tracheal development and for early-stage spermatogenesis [77]. Therefore, HSP60A, HSP60B and HSP60C have distinct functional activities in development, whereas HSP60D is essential for caspase-mediated apoptosis in Drosophila [78]. Our data show that down-regulating the expression of HSP60A, HSP60B or HSP60C led to a partial rescue of the neurodegenerative phenotypes in FUS transgenic flies to various extent, suggesting that these three genes may share common features in FUS proteinopathies. Interestingly, HSP60D down-regulation in FUS transgenic flies did not show any rescue effects, suggesting that caspase-mediated apoptosis might not be critical for neurodegeneration in FUS proteinopathies.

A number of missense mutations have been identified in the human FUS gene among ALS-FUS patients [7,8,14,15,79,80]. In addition, at least four mutations have been detected in the 3’ UTR of the FUS gene among sporadic or familial ALS patients but not in the control samples [16]. Remarkably, fibroblasts from the ALS patients with three different mutations in the 3’UTR showed increased FUS protein expression and accumulation of cytoplasmic FUS [16]. Animal models of FUS proteinopathies established by over-expressing Wt- or ALS-mutant FUS recapitulate major clinical and pathological features of FUS proteinopathies, providing useful systems to study pathogenic mechanisms underlying these devastating diseases [17,18,19,20,21,22,23,24,25]. These results, together with our observation of increased FUS protein in FTLD-FUS patient samples, support the notion that increased FUS expression and cytoplasmic accumulation of FUS likely contribute to the pathogenesis of FUS proteinopathies.

Characteristic cytoplasmic inclusions containing FUS protein have been detected in the affected neural tissues of sporadic ALS (sALS) and FLTD-FUS patients. However, the pathogenic role of FUS and the underlying molecular mechanisms in ALS and FTLD remain to be elucidated. One common finding in ALS and other neurodegenerative diseases is mitochondrial damage [81,82,83,84]. Mitochondrial impairment has been reported in ALS patients as well as animal models for ALS [39,40,43]. Disorganized mitochondria and endoplasmic reticulum have been reported in spinal cord tissue samples from two cases of FUS-positive juvenile ALS patients, including one carrying P525L mutation [46]. In another study, expression of two other ALS-associated FUS mutants, R521G or R521H, was associated with shortened mitochondria in cultured motor neurons *in vitro* [47]. These studies suggested mitochondrial impairment in FUS-positive ALS, although it was not clear whether such mitochondrial defects were a direct consequence of FUS expression or secondary effects following neurodegeneration in ALS. Neither was it clear if mitochondrial defects could be shared pathological features for both ALS-FUS and FTLD-FUS. Our work provides the first EM evidence of mitochondrial localization of FUS not only in cultured cells but also in the FTLD-FUS patient brain samples. Our study provides direct evidence for mitochondrial damage in motor neurons in our animal model for FUS proteinopathies. This is also the first report of mitochondrial damage in FTLD-FUS at the ultra-structural level. Our data obtained from different cellular models and from motor neurons in transgenic flies clearly demonstrate that increased expression of FUS, either Wt- or ALS-mutant, leads to mitochondrial fragmentation, decrease in mitochondrial membrane potential, increased ROS production and eventually neurodegeneration. Our findings suggest that mitochondrial impairments may be an early or initiating event in FUS proteinopathies.

Previous data have shown that certain ALS-associated FUS mutations, such as P525L, increase the cytoplasmic distribution of FUS protein because of the disruption of its nuclear localization signal [17,85]. Interestingly, the P525L-mutation in ALS patients is associated with earlier age-of-onset and faster disease progression (see S1 Table) [7,46,86,87]. Overexpressing either Wt- or ALS-mutant FUS results in a marked increase in mitochondrial fragmentation. In the FUS transgenic flies, FUS expression induced mitochondrial damage and neurodegeneration, both of which were partially rescued by down-regulation of the mitochondrial HSP60 expression. Consistently, brain tissues from at least some FTLD-FUS patients showed increased expression of both FUS and HSP60 proteins.

**Fig 9:**
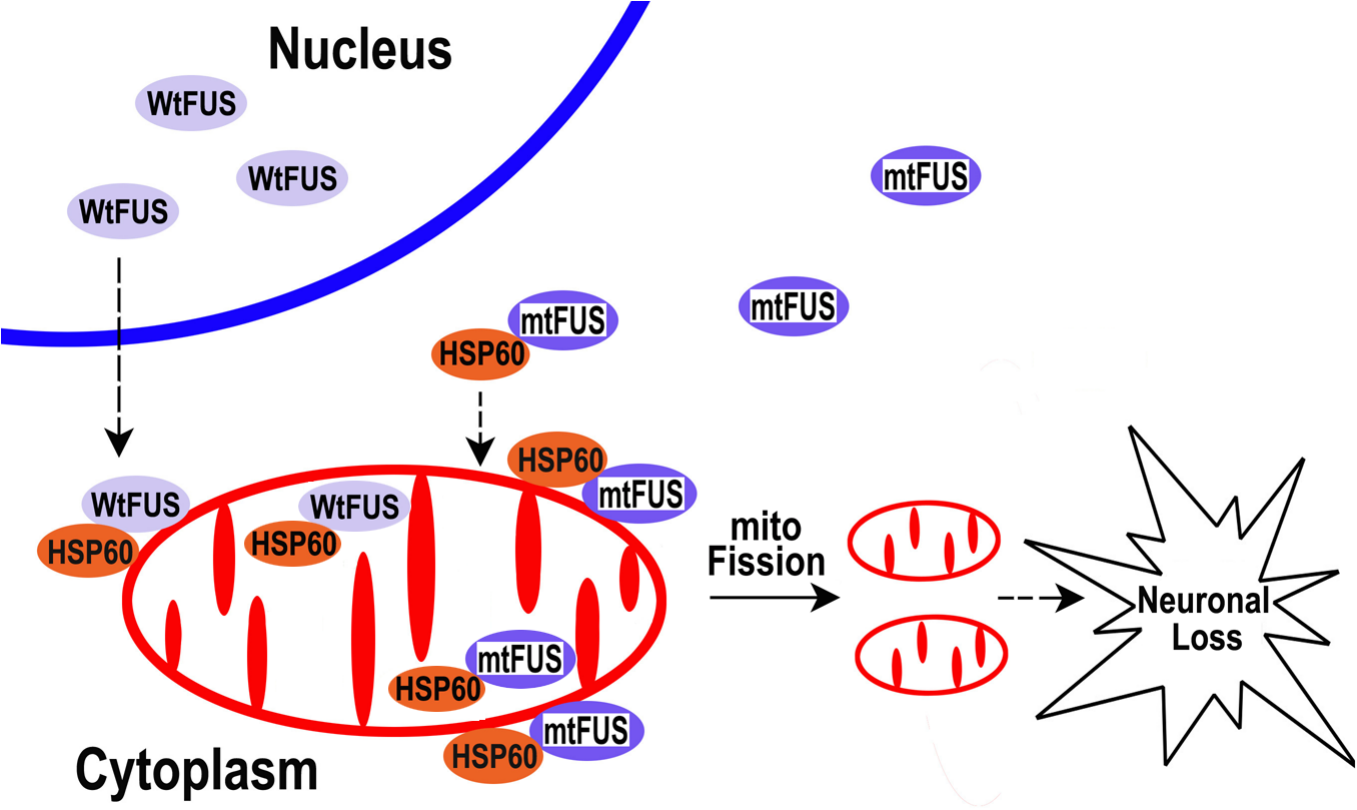
A working model for FUS-induced neurotoxicity. Either an aberrant increase in the expression of Wt-FUS (WtFUS) or FUS mutations that enhance the cytoplasmic redistribution of the mutant FUS protein (mtFUS) leads to abnormal accumulation of cytoplasmic FUS and increased translocation to mitochondria, resulting in excessive mitochondrial fission and damage that eventually culminate in neuronal death.

The data presented in this report led us to propose a working model that can be further tested in our future studies (see Fig 9): increased cytoplasmic FUS protein levels triggered by cellular stresses or pathogenic FUS mutations result in an increased interaction of FUS with mitochondrial chaperone protein HSP60, which promotes FUS localization to mitochondria. Elevated mitochondrial localization of FUS may damage mitochondria, leading to mitochondrial fragmentation. Such mitochondrial impairment may trigger the onset of neuronal damage and neuronal death, eventually resulting in neurodegenerative manifestations of FUS proteinopathies. Recent studies have revealed possible convergence between ALS and FTLD [14]. Our data suggest that one point of such convergence and a critical event in FUS-proteinopathies may be FUS-induced impairment of mitochondria.

Although our data do not rule out the possibility that loss of function caused by sequestration of the wild-type FUS protein into inclusion bodies may also contribute to pathogenesis of FUS-proteinopathies, as previously proposed [88,89], the present study supports the gain-of-function toxicity mechanism. Our work provides strong evidence that mitochondrial damage contributes to FUS-proteinopathies and represents a common molecular pathology shared by ALS-FUS and FTLD-FUS. Moreover, HSP60 signaling pathway may be critical for FUS-induced neurotoxicity, and reducing HSP60 expression or suppressing HSP60 activity may provide therapeutic benefit for FUS-proteinopathies patients with increased HSP60 expression.

## Materials and Methods

### Ethics statement

De-identified postmortem brain samples from patients affected by FTLD-FUS and control subjects were obtained from the Cognitive Neurology &Alzheimer's Disease Center (CNADC) at Northwestern University following institutional and NIH guidelines. All experiments involving animal tissue samples were carried out following institutional and NIH guidelines.

### Fly strains and reagents

Fly strains are as follows: *D42-Gal4 UAS-mitoGFP/TM6B* was from Dr. Y. Zhang [50]. *UAS-Drp1*, *UAS-Drp1-RNAi* and *UAS-Drp1-K38A* were from Dr. B. Lu. *UAS-RFP*, *UAS-Wt-FUS-RFP*, *UAS-P525L-FUS-RFP*were described previously [17]. The HSP60A-RNAi, HSP60B-RNAi, HSP60C-RNAi and HSP60D-RNAi stocks were obtained from Vienna *Drosophila* RNAi Center (VDRC). OK371-Gal4, GMR-Gal4 and Actin5C-Gal4 were obtained from Bloomington Drosophila Stock Center. Flies were raised according to standard procedures at 25°C.

Antibodies used in this study were as follows: rabbit-anti-GFP (Millipore), rabbit-anti-HSP60 (BD) and monoclonal murine anti-myc (Covance), monoclonal murine anti-FUS (ProteinTech Group Inc) and following rabbit polyclonal antibodies against corresponding proteins from ProteinTech Group Inc: CoxIV, Histone H3,TOMM20, MFN2, GAPDH, Actin and PCNA. Anti-RhoA is from Santa Cruz and anti-p637Drp1 is from cell signaling technology (CST).

### Axonal mitochondrial imaging

FUS transgenic and control flies were prepared as described [17]. The third instar larvae were dissected and fixed with 4% paraformaldehyde (PFA, Electron Microscopy Science) for 20 min at room temperature (RT). Following rinse with PBS, larvae were mounted onto coverslips using mounting gel. Confocal images were taken under an Olympus FV1000 confocal microscope.

### Larval movement assay

The assay was done as described [17,90]. Briefly, the larval movement index was measured as the number of peristaltic waves during the period of 2 min in the late third instar larvae expressing control RFP,siHSP60B, P525L-FUS or siHSP60B+P525L-FUS under the OK371-Gal4 driver in a controlled environment (25°C, humidity 50%± 5%, illumination 2800 ±100 lux).

### Immuno-electron microscopy (IEM) or EM

For immuno-EM assay in HEK293 cells, cells stably expressing GFP, Wt- or P525L-mutantas GFP-tagged protein were harvested and fixed with 4% PFA and 0.2% glutaraldehyde (pH7.2) in PBS for 3 hrs at RT. Following rinses and post-fixation processing, gelatin-embedded blocks were prepared in 2.3 M sucrose at 4°C. Ultrathin sections (70-nm) were cut at -120°Cusing dry diamond knives. Following blocking, the sections were immunostained with monoclonal anti-FUS antibody (1:100) and anti-mouse IgG antibody (1:25) conjugated to 10 nm-colloidal gold particles.

For human brain tissue immuno-EM, postmortem frontal cortex samples from control cases and patients affected by FUS-proteinopathies were fixed with 2% PFA and 0.2% glutaraldehyde for 3 hrs at RT. Samples were embedded in 6% gelatin. Immuno-staining was performed as described above.

For EM study of fly eyes, adult fly heads were dissected and fixed in a solution with 4% paraformaldehyde, 2.5% glutaraldehyde in PBS, pH 7.4, for 12 h at 4°C and in a solution with 1% osmium tetroxide in PBS, pH 7.4, for 2 h at room temperature. The tissues were then dehydrated in a series of ethanol solutions (30-min washes in 10, 25, 40, 55, 70, 85 and 100% ethanol) and embedded in spurr resin. Thin sections (70-nm) were prepared and were examined by transmission EM.

All EM images were obtained using a Tecnai Spirit (120kV) or FEI Tecnai 20 electron microscope.

**Scanning electron microscopy (SEM)** was carried out as described before [17].

### Cell cultures and transfection

HEK293T or HT22 cells were cultured (37°C 5% CO2) in DMEM (GIBCO) supplemented with 10% FBS (HyClone or Atlanta Biological). Plasmids were transfected using VigoFect (Vigorous Biotechnology) according to manufacturer’s instructions or calcium phosphate method as described before [91,92]. Cortical neurons were cultured from E15 embryonic mouse brains following the published protocols [93,94].

HEK293-based T-Rex™293 cells (Invitrogen) were transfected with pcDNA4 TO/myc-His plasmids (Invitrogen) expressing either Wt- or P525L-mutant FUS; and stable expressing cells were selected as individual clones in zeocin (400 μg/ml). To induce FUS expression, 0.5 or 1mg/ml tetracycline was added to the culture medium, and cells were incubated for different period of time at 37°C.

The control siRNA and FUS siRNA or HSP60 siRNA were transfected using lipofectamine (Invitrogen) according to manufacturer’s instructions. SiRNA targeting human FUS: 5'-GGACAGCAGCAAAGCTATG-3' [95]. SiRNA targeting human HSP60: 5'-GTGACAAGGCTCAAATTGA-3'.

### Immunoprecipitation

HEK293T cells were used for the transfections and analyses of protein-protein interactions. The experiments were performed at 48 hours post-transfection. The harvested cells were washed with phosphate-buffered saline (PBS) and lysed for 30 minutes on ice in the lysis buffer [91]. The soluble fraction of cell lysates was collected and used for immunoprecipitation with specific antibodies and protein A-agarose (Roche) at 4°C. The immunoprecipitates were examined using Western blotting (WB) with proper antibodies.

### Mitochondrial purification

Mitochondria were purified by percoll gradient ultracentrifugation following published protocols with minor modifications [53,54]. Briefly, stable FUS-expressing HEK cells were lysed in mitochondrial isolation buffer (10mM Tris-MOPS, pH7.4, 1mMEGTA, 250mM sucrose), homogenized with a Glass/Teflon Potter Elvehjem homogenizer (Bellco Glass Inc) and fractionated by sequential centrifugation.

### Cross-linking pull-down assay

After expression of myc-His tagged P525L FUS mutant was induced with 0.5ug/ml tetracycline for 16 hours, cells were briefly washed with PBS containing 1mM MgCl_2_ (PBS, MgCl_2_), and treated with 1% PFA in PBS MgCl_2_ for 10 minutes. Cells were washed with 50mM Tris-HCl buffer (pH 7.4) containing 120mM NaCl and 1mM MgCl_2_ twice and treated with the same buffer for 10 min. Cells were washed with PBS- MgCl_2_ once again and lysed with phosphate buffer (pH 8.0) containing 1% NP-40, 200mM NaCl and 1mM PMSF. Soluble fractions were obtained by centrifugation at 2500g for 30 min and twice volume of 6M guanidine HCl in 50mM phosphate buffer (pH 8.0) was added. FUS proteins were collected with Ni-NTA agarose, washed with 6M guanidine HCl in 50mM phosphate buffer (pH 8.0) twice, and then eluted with 100mM PIPES (pH 6.6) containing 0.1% SDS, 5mM EDTA. The obtained solutions were diluted ten times with 20mM HEPES (pH 7.4) containing 0.5% Triton X-100, 0.05% deoxycholate, 100mM NaCl, 5% glycerol and 0.5mM PMSF to carry out immunoprecipitation with anti-myc antibody and protein A/G beads. Immunoprecipitated beads were resuspended with 6M guanidine HCl in 50mM phosphate buffer (pH 8.0), and FUS-myc-His protein was pull-down with Ni-NTA beads (by repurification with Ni-NTA, antibody can be removed so that background signals can be eliminated in WB analyses). Pull-down fractions were analyzed by Western blotting.

### GST pull-down assay

GST pull-down assay was performed as described by [96]. GST tagged Wt or P525L or fragments FUS protein were expressed in bacteria and purified using glutathione 4B Sepharose beads. His tagged HSP60 were similarly expressed in bacteria and purified using nickel beads. Purified GST, or Wt, P525L, fragments FUS-GST was incubated with purified HSP60-His in TNE buffer (10 mM Tris, pH 8.0, 150 mM NaCl, 1 mM EDTA, 1% NP-40) on ice for 1 hour. Then, supernatants were incubated with glutathione 4B Sepharose beads at 4°C for 3 hours. The glutathione beads were then washed extensively with ice-cold TNE buffer, and bound proteins were subjected to SDS-PAGE followed by immunoblotting analysis.

### RNaseA treatment

The RNaseA treatment assay was performed as [97]. Briefly, the purified protein were treated with 50ug/ml RNaseA for 30 min at 37°C. Then GST pull-down assay was performed as previous described.

### Measurement of mitochondrial membrane potential

Mitochondrial membrane potential was measured by staining cells with tetramethylrhodamine methyl ester (TMRM) (Invitrogen). HEK293 cells were transfected with the GFP vector control, Wt- or P525L-mutant FUS as GFP-tagged proteins. Cells were incubated with 20 nM TMRM for 20 min at 37°C, 19 hrs post-transfection. Cells were washed three times with PBS and cultured in opti-MEM (without phenol red) supplemented with 10% FBS and 5 nM TMRM. Confocal images were taken using an Olympus FV1000 microscope. The TMRM intensity in different groups was measured by FACS (BD FACS AriaII) and analyzed using FlowJo software. Data were obtained from four independent experiments, and 2000 cells were examined per group in each experiment.

### Mitochondrial ROS measurement

Mitochondrial ROS production was measured using mitoSOX-Red (Invitrogen). HEK293 cells were transfected with plasmids expressing either the GFP vector control, Wt or P525L FUS-GFP. 24 hours post-transfection, cells were stained with 5μM mitoSOX-Red for 20 min at 37°C. After washes, cells were fixed with 4% PFA for 20 min at room temperature followed by FACS analysis with mitoSOX-Red fluorescence intensity determined using FlowJo software. Confocal images were taken using an Olympus FV1000 microscope.

### RT-PCR

To measure HSP60B expression levels in the control and siRNA flies, total RNA was prepared from flies using Trizol (Invitrogen), reverse transcribed, and subjected to PCR analysis. For HSP60B, PCR (25 cycles) was carried out using the following primers: 5’- CTGAGGATGCCTTGCCAGACC-3’ and 5’- GCAGCACCTTTGTGGGATCAATA-3’. For Actin, PCR analysis (21 cycles) using the specific primers: 5’-GAGCGCGGTTACTCTTTCAC-3’ and 5’-ATCCCGATCCTGATCCTCTT-3’.

### Quantitative analyses of mitochondrial morphology

Mitochondria from HT22 cells or cortical neurons were quantified as [98]. Briefly, the percentage of cells showed tubular, fragmented or intermediate pattern was counted and statistic analysis. Mitochondria from fly motor neuron were measured their length by using Image J. The mitochondrial length from Wt- or P525L-mutant FUS groups was normalized to the control (Ctr) group and presented as the percentage of the mitochondrial size in the control group.

Mitochondrial size in EM analyses were quantified by their cross sectional areas by using Image J software. The mitochondrial size from Wt- or P525L-mutant group was normalized to the control group and presented as percentage of the mitochondrial size in the control group. Normal or abnormal cristae morphology of mitochondria was defined as previously published [99,100]. Briefly, normal mitochondria with numerous well-organized cristae or abnormal mitochondria showing ring structures or loss of cristae were scored and quantified as the percentage of total mitochondria examined.

**Statistical analyses** were performed using either one-way ANOVA, followed by student t-test, Chi-square test or Bonferroni multiple comparison for comparing individual groups, as indicated in corresponding figure legends. The bar graphs with error bars represent mean ± standard error of the mean (SEM). Significance is indicated by asterisks: *, *P* < 0.05; **, *P*< 0.01; ***, *P*< 0.001.

## Supporting Information

S1 Table The age of onset and duration of ALS-FUS (published) and FTLD-FUS (this study) patients. (DOCX)

S1 Fig Western blotting and IEM assays demonstrating specificity of the FUS antibody. (A) A monoclonal antibody (ProteinTech Group Inc, USA) detected a single band in the total cell lysates prepared from HEK293 cells (marked by the arrow), and this band is almost eliminated when the antibody was pre-absorbed (FUS-absorbed) in the presence of purified recombinant FUS protein. (B)The immunoEM (IEM) signals were almost reduced to the background level when FUS-absorbed antibody was used in staining the human brain tissue samples. The number of 10nm-gold particles per square μm of the brain section was quantified in the fronto-cortical tissues from postmortem brain of FTLD-FUS case #C (left) or control case #3 (right). All data were analyzed using one-way ANOVA (n = 7, ***: p<0.0001). (C) Knocking down FUS by specific siRNA significantly reduced the FUS-specific Western blotting band signals. Two independent knocking-down experiments were carried out (shown in lanes 1–4). (D) Quantification of WB signals shown in panel C. (E) Western-blotting images of brain lysates from the wild-type (Wt; +/+), heterozygous (Het; +/-) and homozygous deficient-knockout (KO;-/-) FUS mice.
(TIF)

S2 Fig Increased expression of FUS alters mitochondrial protein levels. (A, B) HEK293T cells were transfected with GFP, Wt-FUS-GFP or P525L-FUS-GFP, and cell lysates were subjected to Western blotting analyses 24 hrs post-transfection. (C-E) Quantification of protein levels as indicated. Data were analyzed using one-way ANOVA (n>3, *: p<0.05).
(TIF)

S3 Fig Expression of wild-typeDrp1 or K38A-Drp1 in primary cortical neurons expressing Wt- or P525L- mutant FUS and in FUS transgenic flies. (A) The mitoRed plasmid was co-transfected together with plasmids expressing either the control vector (Ctr), or Wt- or K38A-Drp1 into E18 murine cortical neurons expressing P525L FUS. Arrow marks tubular mitochondria, arrowhead marks fragmented mitochondria, “*” marks condensed or fragmented nuclei (signs of cell death). Insets show the boxed areas at a higher magnification. (B) Quantification of the percentage of cells containing fragmented mitochondria. (C) Quantification of the percentage of cells with condensed nuclei. All data were analyzed using one-way ANOVA with Bonferroni post-test (***: p<0.0001). (D) Light microscopic images of eyes of control or Drp1 or siDrp1 or K38A-Drp1 flies. Fly genotypes: Ctr: GMR-Gal4/UAS-Wt-FUS-RFP or GMR-Gal4/UAS-P525L-FUS-RFP; Drp1: GMR-Gal4/UAS-Wt-FUS-RFP/UAS-Drp1 or GMR-Gal4/UAS-P525L-FUS-RFP/UAS-Drp1; siDrp1: GMR-Gal4/UAS-Wt-FUS-RFP/UAS-siDrp1 or GMR-Gal4/UAS-P525L-FUS-RFP/UAS-siDrp1; K38A-Drp1: GMR-Gal4/UAS-Wt-FUS-RFP/UAS-K38A-Drp1 or GMR-Gal4/UAS-P525L-FUS-RFP/UAS-K38A-Drp1. (E) Scanning electron microscopic images of flies expressing P525L FUS with control or co-expressing K38A-Drp1, with the right panels showing higher magnification images of the corresponding ones.
(TIF)

S4 Fig HSP60 interacts with FUS and mediates FUS mitochondrial localization. (A) The purified proteins were treated with RNaseA (50ug/ml) for 30 min at 37°C. GST pull-down was performed and bound proteins were analyzed by Western blotting (WB). The lower panel shows Coomassie blue staining of GST fusion proteins used in the assay. (B) A darker exposure of panel 5 and 6 in Fig 6D shows multiple bands including 70 and 130kDa in the cross-linked cell lysates using anti-FUS antibody. (C) Western blotting (WB) analysis of nuclear or cytosolic fractions from Fig 6E to show endogenous FUS localization. Histone H3 was used as a nuclear marker and GAPDH was used as a cytosolic marker. The nuclear FUS levels were increased when HSP60 expression was knocked down by siHSP60. (D) Quantification of the HSP60 levels in the nuclear fractions and in the cytosolic fractions. (E) Quantification of FUS levels in the nuclear fractions and the cytosolic fractions.(F)P525L-FUS-expressing stable HEK cells were transfected with the control or HSP60 siRNAs and harvested for mitochondrial purification 72-hr post-transfection. The mitochondrial purity was confirmed by the enrichment of mitochondrial protein TOM20 and the absence of cytoplasmic proteins such as RhoA or nuclear protein PCNA. The mitochondrial levels of the P525L-mutant or the endogenous Wt- FUS were decreased when HSP60 expression was down-regulated by siHSP60, as shown by WB. (G) Quantification of the HSP60 levels in the total cell extracts and in the mitochondrial fractions. (H) Quantification of FUS levels in total cell extracts and the mitochondrial fractions. All data were analyzed using one-way ANOVA with Bonferroni post-test (*: p<0.05; **: p<0.01; ***: p<0.0001).
(TIF)

S5 Fig Knocking down HSP60 partially rescued FUS-induced retinal generation phenotype without altering the level of FUS expression. (A) Western blotting experiments using the cell lysates prepared from fly heads in corresponding fly groups demonstrate that siHSP60 expression did not affect the level of FUS transgene expression (at least 30 fly heads were used in each group). Beta-actin was used as an internal control for total protein loaded. (B) Light microscopic images of fly eyes in the control or siHSP60A or siHSP60B, siHSP60C or siHSP60D groups. Arrows mark the retinal areas with improved ommatidial organization and reduced retinal degeneration when siHSP60A, or siHSP60B or siHSP60C was expressed in photoreceptor cells of the Wt- or P525L-mutant FUS transgenic flies. Fly genotypes: Ctr: GMR-Gal4/UAS-Wt-FUS-RFP or GMR-Gal4/UAS-P525L-FUS-RFP; siHSP60A: GMR-Gal4/UAS-Wt-FUS-RFP/UAS-siHSP60A or GMR-Gal4/UAS-P525L-FUS-RFP/UAS- siHSP60A; siHSP60B: GMR-Gal4/UAS-Wt-FUS-RFP/UAS-siHSP60B or GMR-Gal4/UAS-P525L-FUS-RFP/UAS- siHSP60B; siHSP60C: GMR-Gal4/UAS-Wt-FUS-RFP/UAS-siHSP60C or GMR-Gal4/UAS-P525L-FUS-RFP/UAS- siHSP60C; siHSP60D: GMR-Gal4/UAS-Wt-FUS-RFP/UAS-siHSP60D or GMR-Gal4/UAS-P525L-FUS-RFP/UAS- siHSP60D.
(TIF)

S6 Fig A phylogram showing the evolutionary conservation between the four *HSP60* homolog genes in *Drosophila Melanogaster* (*Dm*) and the *HSP60* gene in *Homo Sapiens* (*Hs*). The phylogram was generated by ClustalW alignment of amino acid sequences, showing the predicted relationship between four *DmHSP60* genes and the *HsHSP60* gene.
(TIF)

S7 Fig HSP60B expression in the control and HSP60B-siRNA flies. (A) RT-PCR analysis of the control and siHSP60B flies using specific primers to determine the expression levels of HSP60B. Actin expression was used as an internal control. (B) Quantification of the HSP60B expression levels in respective groups. Data were collected from 3 independent experiments and analyzed using two-tailed t-test (*: p<0.05). (C) Light microscopic images of eyes of control or siHSP60B. No morphological changes were detected in HSP60B-knock down flies.
(TIF)

S8 Fig TEM sections of fly retina at day 15 and quantification of nuclear size, Rhabdomere (Rh) number, mitochondrial size in fly retina. (A) At day 15, marked photoreceptor cell loss is detected in flies expressing Wt- or P525L-mutant FUS but not in the control flies (scale bar: 5um). (B) The photoreceptors in flies expressing Wt- or P525L-mutant FUS show nuclei of increased size as compared to the control group (scale bar: 1um). (C) Quantification of nucleic size in the corresponding fly photoreceptors (n≥16). (D) Quantification of the number of remaining rhabdomeres (including fragments of Rh, n≥12 for each group). (E) More than 100 mitochondria in each group were quantified using Image J. All data were analyzed using one-way ANOVA with Bonferroni post-test (*: p<0.05; **:p<0.01; ***: p<0.0001).
(TIF)
