# Supplementary material for: FUS Interacts with HSP60 to Promote Mitochondrial Damage
Source: PLoS Genet. 2015 Sep 3;11(9):e1005357. doi: 10.1371/journal.pgen.1005357 (PMC4559378; doi:10.1371/journal.pgen.1005357)
Supplement: S1 Table — (DOCX) [file pgen.1005357.s001.docx]

**Supplementary Table for:**

**FUS interacts with HSP60 to promote mitochondrial damage**

Jianwen Deng^1,5^, Mengxue Yang^1,2,5^, Yanbo Chen^2, 3^, Xiaoping Chen^2^, Jianghong Liu^1^, Shufeng Sun^1^, Haipeng Cheng^2^, Yang Li^2,4^, Eileen H. Bigio^6^, Marsel Mesulam^6^, Qi Xu^3^, Sidan Du^4^, Kazuo Fushimi^2^, Li Zhu^1,*^ and Jane Y. Wu^1,2,*^

| **Table S1. The age of onset and duration of ALS-FUS (published) and FTLD-FUS (this study) patients.** | | | | | | |
| --- | --- | --- | --- | --- | --- | --- |
|  |  | FUS Mutations  mutation | Subject | Age of Onset  (years) | Duration (Months)  (months) | Reference |
| ALS-FUS | Sporadic | P525L | 1 | 13 | 20 | [46] |
|  |  | P525L | 2 | 22 | 10 | [86] |
|  |  | P525L | 3 | 18 | 11 |  |
|  | Familial | P525L | 1 | 21 | <12 | [87] |
|  |  | P525L | 2 | 32 | <12 |  |
|  |  | P525L | 3 | 16 | <12 |  |
|  |  | P525L | 4 | 27 | <24 |  |
|  |  | P525L | 5 | 22 | 6 | [7] |
|  | Familial | R524S | 1 | 34 | 39 | [7] |
| FTLD-FUS | Sporadic | None detected | A | 33 | 96 | This study |
|  | Sporadic | None detected | B | 58 | 72 | This study |
|  | Sporadic | None detected | C | 48 | 84 | This study |
