# Supplementary figures and images for: FUS Interacts with HSP60 to Promote Mitochondrial Damage
Source: PLoS Genet. 2015 Sep 3;11(9):e1005357. doi: 10.1371/journal.pgen.1005357 (PMC4559378; doi:10.1371/journal.pgen.1005357)

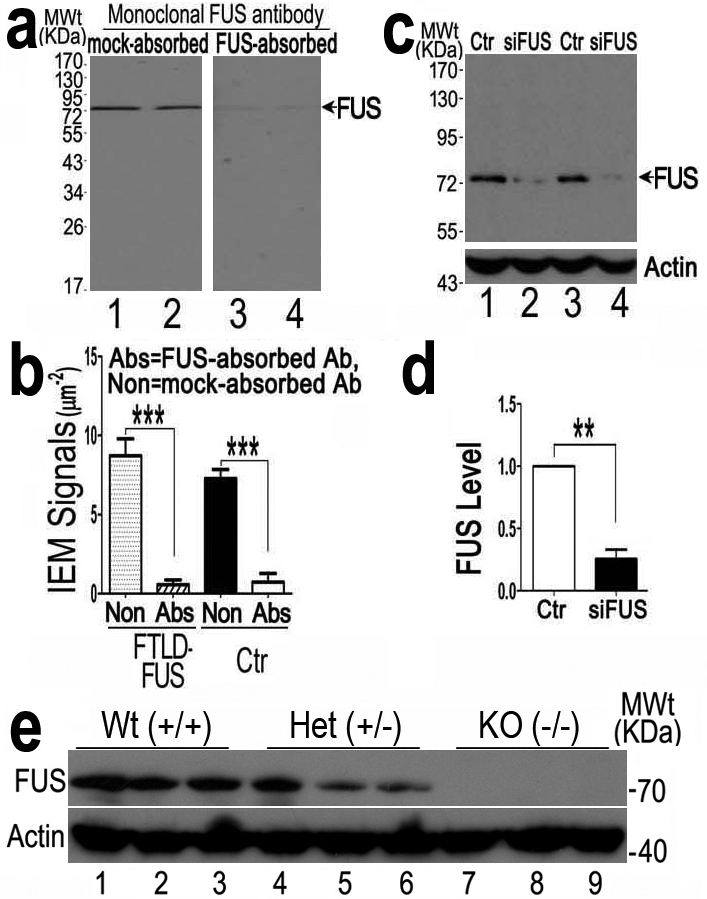

Supplement: S1 Fig — (A) A monoclonal antibody (ProteinTech Group Inc, USA) detected a single band in the total cell lysates prepared from HEK293 cells (marked by the arrow), and this band is almost eliminated when the antibody was pre-absorbed (FUS-absorbed) in the presence of purified recombinant FUS protein. (B)The immunoEM (IEM) signals were almost reduced to the background level when FUS-absorbed antibody was used in staining the human brain tissue samples. The number of 10nm-gold particles per square μm of the brain section was quantified in the fronto-cortical tissues from postmortem brain of FTLD-FUS case #C (left) or control case #3 (right). All data were analyzed using one-way ANOVA (n = 7, ***: p<0.0001). (C) Knocking down FUS by specific siRNA significantly reduced the FUS-specific Western blotting band signals. Two independent knocking-down experiments were carried out (shown in lanes 1–4). (D) Quantification of WB signals shown in panel C. (E) Western-blotting images of brain lysates from the wild-type (Wt; +/+), heterozygous (Het; +/-) and homozygous deficient-knockout (KO;-/-) FUS mice. (TIF) [file pgen.1005357.s002.tif]

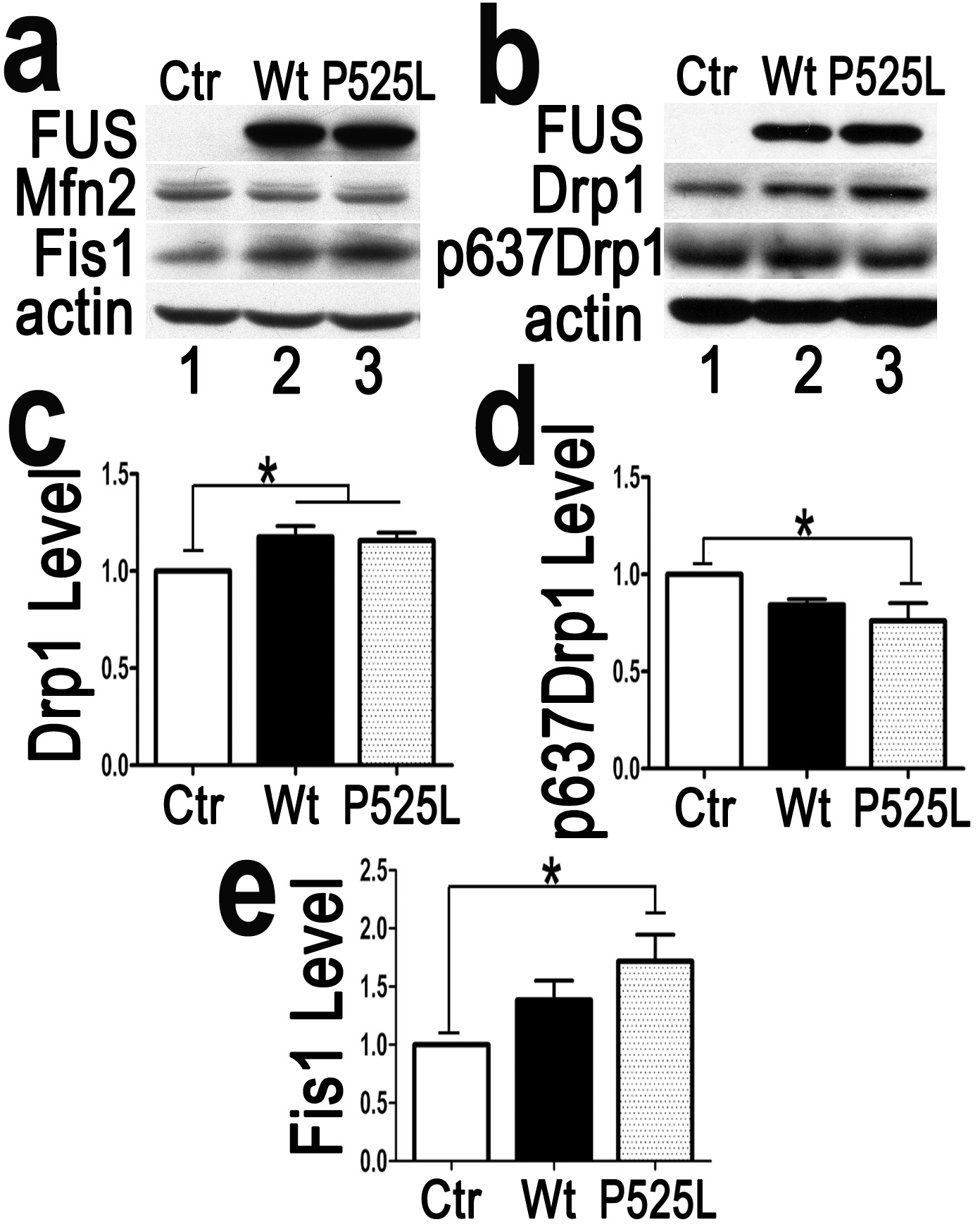

Supplement: S2 Fig — (A, B) HEK293T cells were transfected with GFP, Wt-FUS-GFP or P525L-FUS-GFP, and cell lysates were subjected to Western blotting analyses 24 hrs post-transfection. (C-E) Quantification of protein levels as indicated. Data were analyzed using one-way ANOVA (n>3, *: p<0.05). (TIF) [file pgen.1005357.s003.tif]

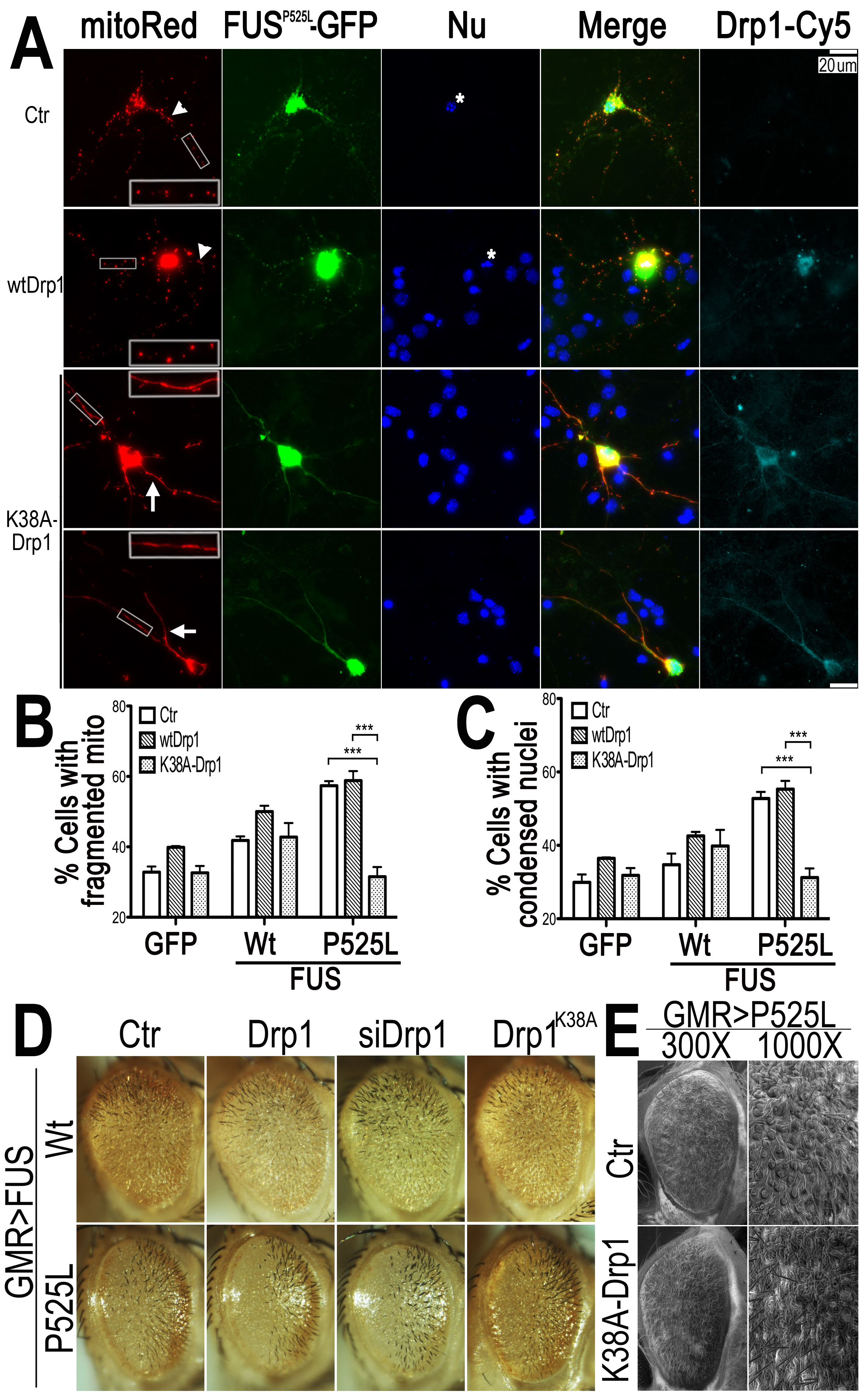

Supplement: S3 Fig — (A) The mitoRed plasmid was co-transfected together with plasmids expressing either the control vector (Ctr), or Wt- or K38A-Drp1 into E18 murine cortical neurons expressing P525L FUS. Arrow marks tubular mitochondria, arrowhead marks fragmented mitochondria, “*” marks condensed or fragmented nuclei (signs of cell death). Insets show the boxed areas at a higher magnification. (B) Quantification of the percentage of cells containing fragmented mitochondria. (C) Quantification of the percentage of cells with condensed nuclei. All data were analyzed using one-way ANOVA with Bonferroni post-test (***: p<0.0001). (D) Light microscopic images of eyes of control or Drp1 or siDrp1 or K38A-Drp1 flies. Fly genotypes: Ctr: GMR-Gal4/UAS-Wt-FUS-RFP or GMR-Gal4/UAS-P525L-FUS-RFP; Drp1: GMR-Gal4/UAS-Wt-FUS-RFP/UAS-Drp1 or GMR-Gal4/UAS-P525L-FUS-RFP/UAS-Drp1; siDrp1: GMR-Gal4/UAS-Wt-FUS-RFP/UAS-siDrp1 or GMR-Gal4/UAS-P525L-FUS-RFP/UAS-siDrp1; K38A-Drp1: GMR-Gal4/UAS-Wt-FUS-RFP/UAS-K38A-Drp1 or GMR-Gal4/UAS-P525L-FUS-RFP/UAS-K38A-Drp1. (E) Scanning electron microscopic images of flies expressing P525L FUS with control or co-expressing K38A-Drp1, with the right panels showing higher magnification images of the corresponding ones. (TIF) [file pgen.1005357.s004.tif]

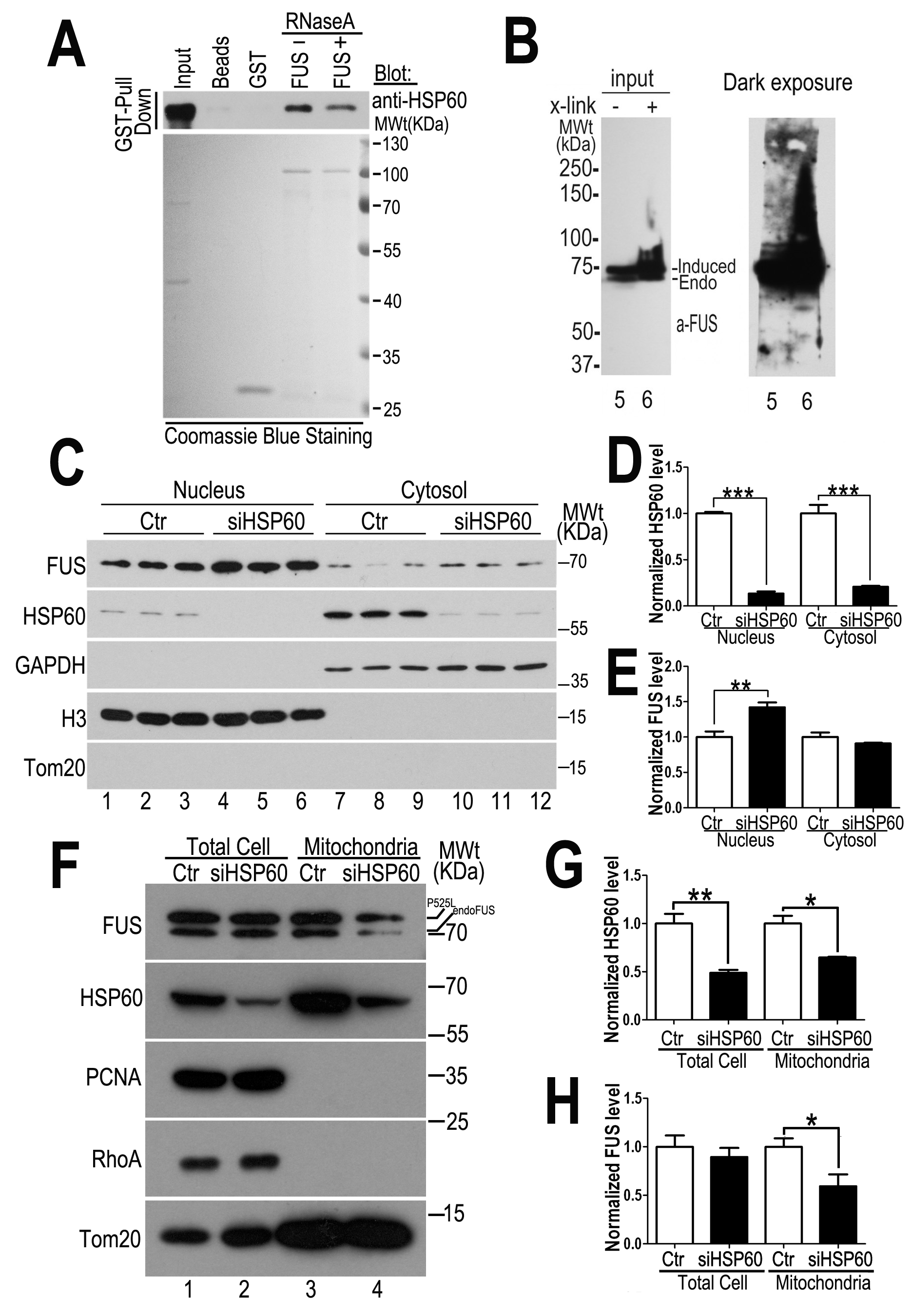

Supplement: S4 Fig — (A) The purified proteins were treated with RNaseA (50ug/ml) for 30 min at 37°C. GST pull-down was performed and bound proteins were analyzed by Western blotting (WB). The lower panel shows Coomassie blue staining of GST fusion proteins used in the assay. (B) A darker exposure of panel 5 and 6 in Fig 6D shows multiple bands including 70 and 130kDa in the cross-linked cell lysates using anti-FUS antibody. (C) Western blotting (WB) analysis of nuclear or cytosolic fractions from Fig 6E to show endogenous FUS localization. Histone H3 was used as a nuclear marker and GAPDH was used as a cytosolic marker. The nuclear FUS levels were increased when HSP60 expression was knocked down by siHSP60. (D) Quantification of the HSP60 levels in the nuclear fractions and in the cytosolic fractions. (E) Quantification of FUS levels in the nuclear fractions and the cytosolic fractions.(F)P525L-FUS-expressing stable HEK cells were transfected with the control or HSP60 siRNAs and harvested for mitochondrial purification 72-hr post-transfection. The mitochondrial purity was confirmed by the enrichment of mitochondrial protein TOM20 and the absence of cytoplasmic proteins such as RhoA or nuclear protein PCNA. The mitochondrial levels of the P525L-mutant or the endogenous Wt- FUS were decreased when HSP60 expression was down-regulated by siHSP60, as shown by WB. (G) Quantification of the HSP60 levels in the total cell extracts and in the mitochondrial fractions. (H) Quantification of FUS levels in total cell extracts and the mitochondrial fractions. All data were analyzed using one-way ANOVA with Bonferroni post-test (*: p<0.05; **: p<0.01; ***: p<0.0001). (TIF) [file pgen.1005357.s005.tif]

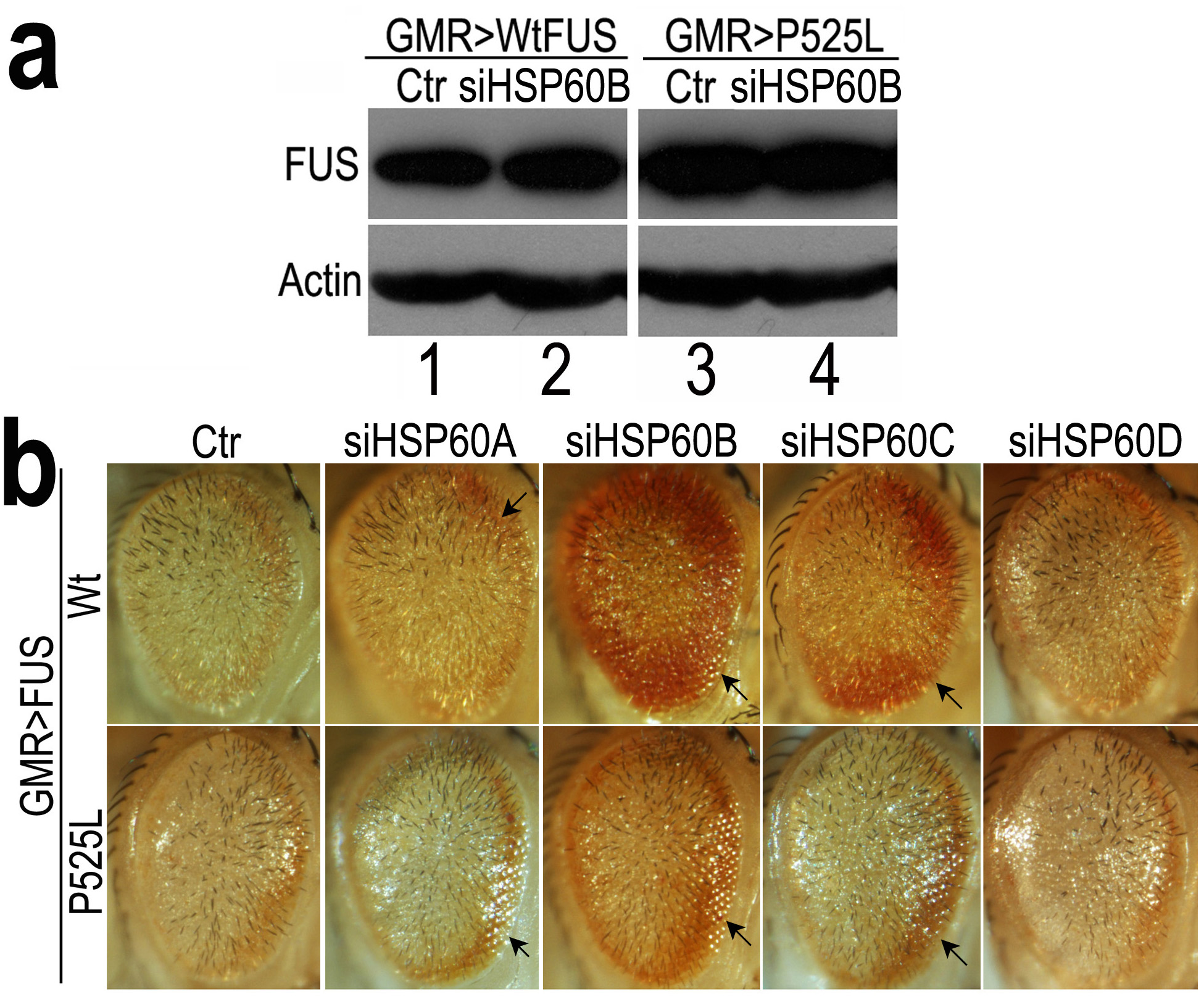

Supplement: S5 Fig — (A) Western blotting experiments using the cell lysates prepared from fly heads in corresponding fly groups demonstrate that siHSP60 expression did not affect the level of FUS transgene expression (at least 30 fly heads were used in each group). Beta-actin was used as an internal control for total protein loaded. (B) Light microscopic images of fly eyes in the control or siHSP60A or siHSP60B, siHSP60C or siHSP60D groups. Arrows mark the retinal areas with improved ommatidial organization and reduced retinal degeneration when siHSP60A, or siHSP60B or siHSP60C was expressed in photoreceptor cells of the Wt- or P525L-mutant FUS transgenic flies. Fly genotypes: Ctr: GMR-Gal4/UAS-Wt-FUS-RFP or GMR-Gal4/UAS-P525L-FUS-RFP; siHSP60A: GMR-Gal4/UAS-Wt-FUS-RFP/UAS-siHSP60A or GMR-Gal4/UAS-P525L-FUS-RFP/UAS- siHSP60A; siHSP60B: GMR-Gal4/UAS-Wt-FUS-RFP/UAS-siHSP60B or GMR-Gal4/UAS-P525L-FUS-RFP/UAS- siHSP60B; siHSP60C: GMR-Gal4/UAS-Wt-FUS-RFP/UAS-siHSP60C or GMR-Gal4/UAS-P525L-FUS-RFP/UAS- siHSP60C; siHSP60D: GMR-Gal4/UAS-Wt-FUS-RFP/UAS-siHSP60D or GMR-Gal4/UAS-P525L-FUS-RFP/UAS- siHSP60D. (TIF) [file pgen.1005357.s006.tif]

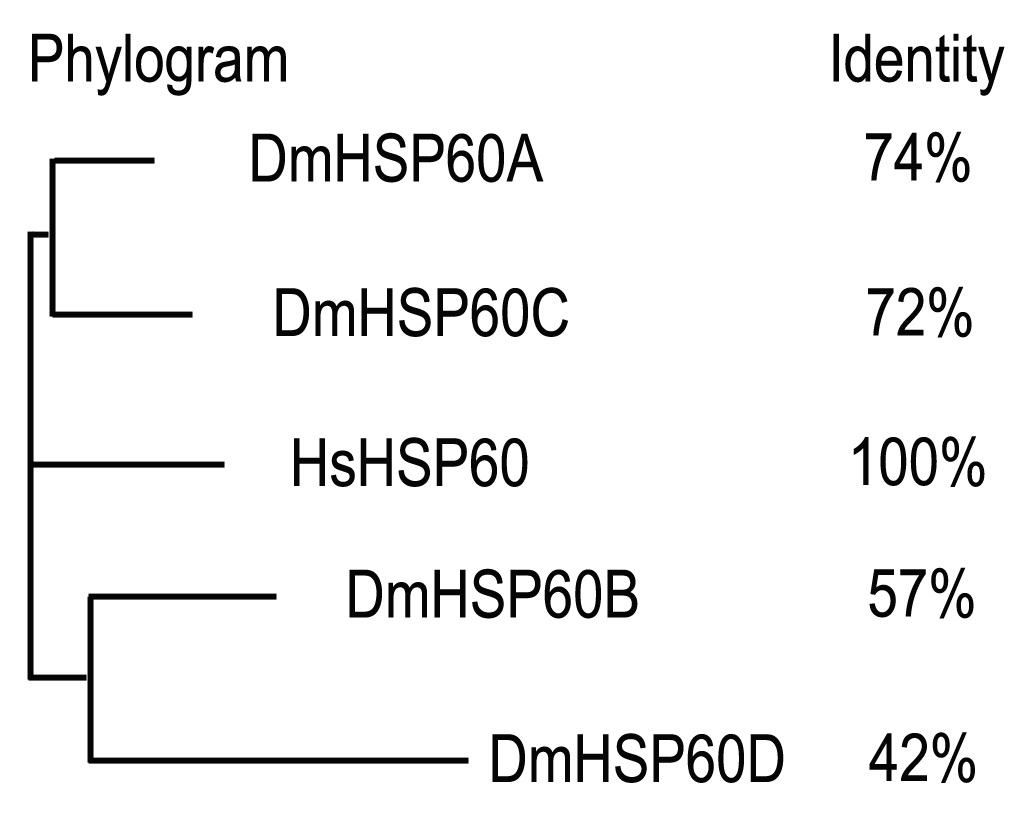

Supplement: S6 Fig — The phylogram was generated by ClustalW alignment of amino acid sequences, showing the predicted relationship between four DmHSP60 genes and the HsHSP60 gene. (TIF) [file pgen.1005357.s007.tif]

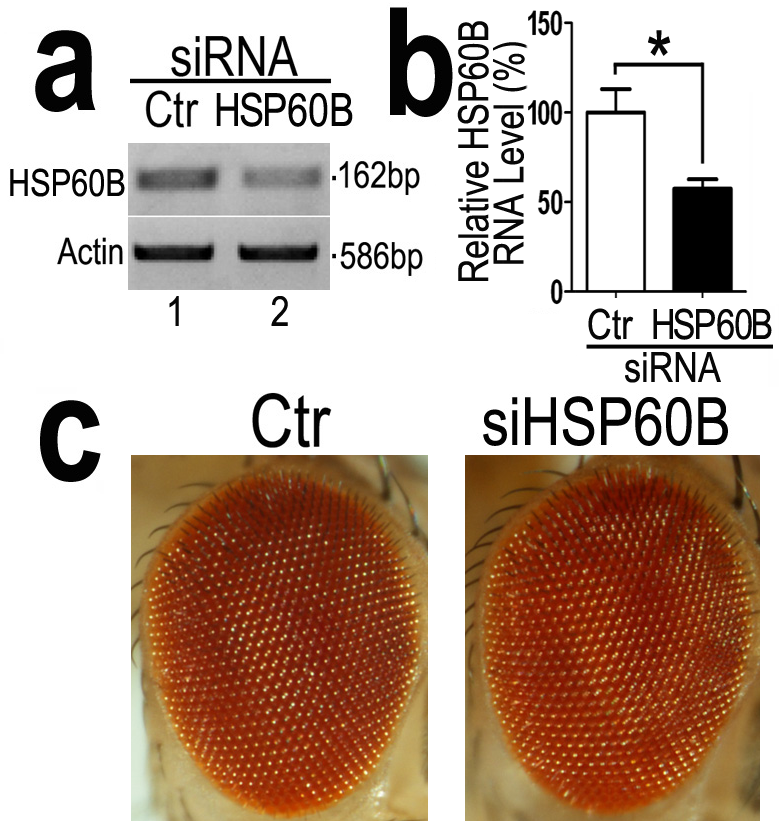

Supplement: S7 Fig — (A) RT-PCR analysis of the control and siHSP60B flies using specific primers to determine the expression levels of HSP60B. Actin expression was used as an internal control. (B) Quantification of the HSP60B expression levels in respective groups. Data were collected from 3 independent experiments and analyzed using two-tailed t-test (*: p<0.05). (C) Light microscopic images of eyes of control or siHSP60B. No morphological changes were detected in HSP60B-knock down flies. (TIF) [file pgen.1005357.s008.tif]

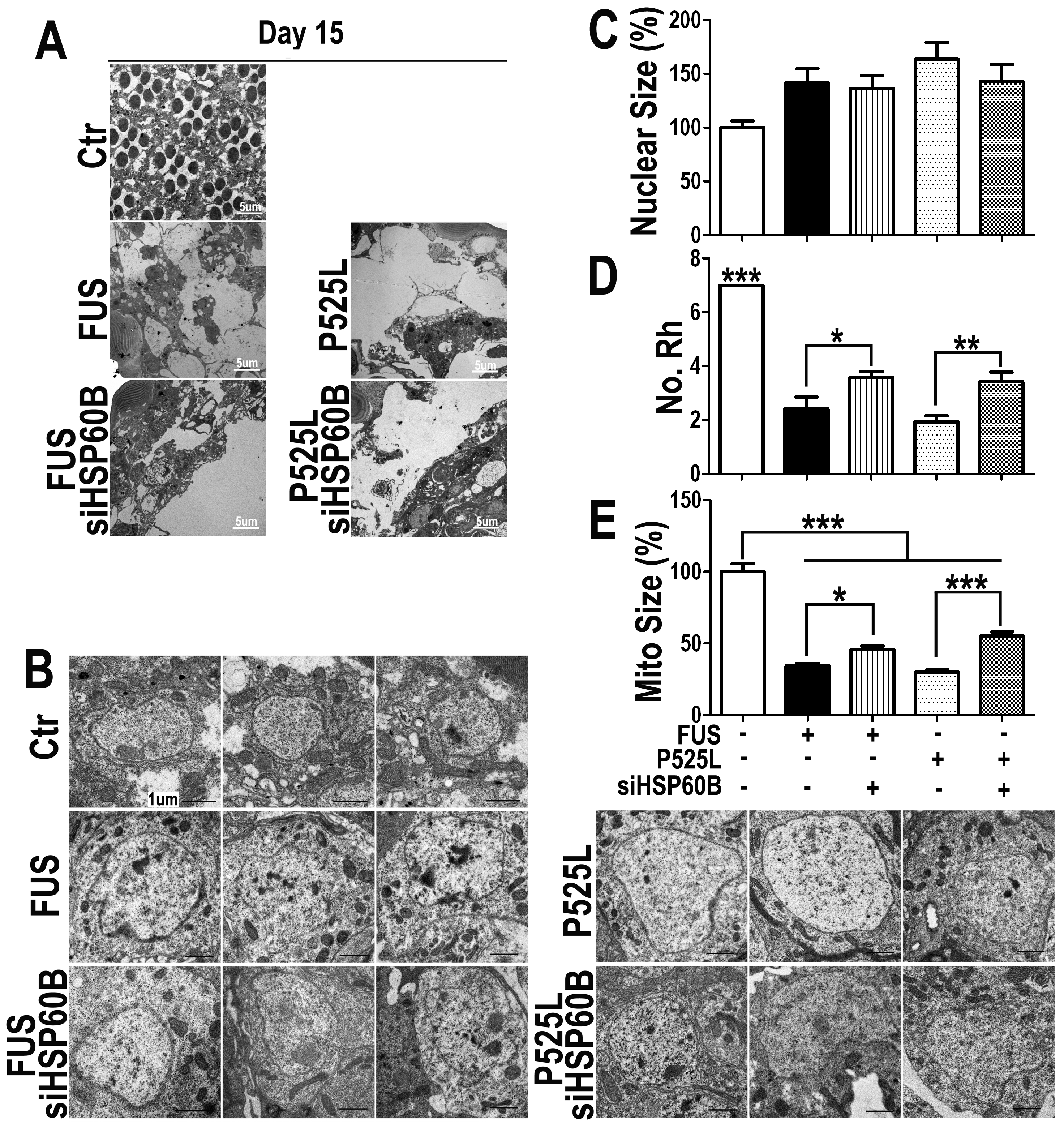

Supplement: S8 Fig — (A) At day 15, marked photoreceptor cell loss is detected in flies expressing Wt- or P525L-mutant FUS but not in the control flies (scale bar: 5um). (B) The photoreceptors in flies expressing Wt- or P525L-mutant FUS show nuclei of increased size as compared to the control group (scale bar: 1um). (C) Quantification of nucleic size in the corresponding fly photoreceptors (n≥16). (D) Quantification of the number of remaining rhabdomeres (including fragments of Rh, n≥12 for each group). (E) More than 100 mitochondria in each group were quantified using Image J. All data were analyzed using one-way ANOVA with Bonferroni post-test (*: p<0.05; **:p<0.01; ***: p<0.0001). (TIF) [file pgen.1005357.s009.tif]
